# Development of Novel Pegylated Imidazobenzodiazepines Targeting α5 GABA_A_ Receptors for Anxiety: A Comparative Prodrug and Metabolite Study

**DOI:** 10.1021/acschemneuro.6c00222

**Published:** 2026-08-17

**Authors:** Mubaraq A. Toriola, Ethan T. Kowalczyk, Kayode M. Medubi, Michelle J. Meyer, Maya R. T. Fernando, Petra Scholze, Nethyanji Premananda, Shaun G. Harrington, Braden R. Barkley, Daniel A. Webb, Leggy A. Arnold

**Affiliations:** † Department of Chemistry and Biochemistry and the Milwaukee Institute for Drug Discovery, 14751 University of Wisconsin-Milwaukee, 2000 E. Kenwood Blvd, Milwaukee, Wisconsin 53211, United States; ‡ Department of Pathobiology of the Nervous System, Center for Brain Research, 31235 Medical University of Vienna, Spitalgasse 4, Vienna 1090, Austria

**Keywords:** pegylation, GABA(A) receptor, anxiety, metabolism, imidazobenzodiazepines

## Abstract

We report on the development of a highly water-soluble pegylated imidazodiazepine ETK-II-83. The compound binds selectively to GABA_A_ receptors among forty-five other receptors expressed in the brain with a higher affinity for the α5-containing GABA_A_ receptors. The compound is converted *in vitro* by liver microsomes into ETK-III-9. The metabolic reaction selectively oxidizes the polyethylene glycol groups of ETK-II-83 forming eventually the stable 2-hydroxyethyl amide-bearing ETK-III-9. The conversion is fast with a half-life of 5.33 min. ETK-III-9 strongly binds to α3/α5 containing GABA_A_ receptors. A pharmacokinetic study with ETK-II-83 confirmed metabolic instability resulting in low AUCs for blood and brain. Interestingly, higher brain concentrations than blood concentrations (K_P_ = 5.17) were observed. The in vivo formation of ETK-III-9 inside ETK-II-83-treated animals was rapid and peaked at 40 min. Almost equal amounts of ETK-III-9 were found in blood and brain with half-lives of 219 and 202 min, respectively. We also conducted a pharmacokinetic study with ETK-III-9 at the same dose and observed three times higher AUCs for blood and brain. Importantly, the free brain concentration of ETK-III-9 in ETK-II-83-administrated animals was higher than its affinity for the GABA_A_ receptors. This was confirmed by investigating ETK-II-83 with two anxiety mouse models. For the elevated plus maze, ETK-III-9- and ETK-II-83-treated animals spent significantly more time in the open arm than vehicle-treated animals, without any differences in the overall distance traveled. ETK-III-9- and ETK-II-83-treated animals also buried fewer marbles than vehicle control animals. To demonstrate that these effects were not caused by sedation or inhibition of sensorimotor inhibition, we conducted open field and rotarod tests. For both tests, ETK-III-9- and ETK-II-83-treated animals did not behave differently from vehicle-treated mice in contrast to diazepam-treated animals. The innovation is a highly water-soluble prodrug ETK-II-83 that metabolizes to ETK-III-9, an α3/α5 GABA_A_ receptor-selective compound that reduces anxiety in rodent models.

## Graphical abstract

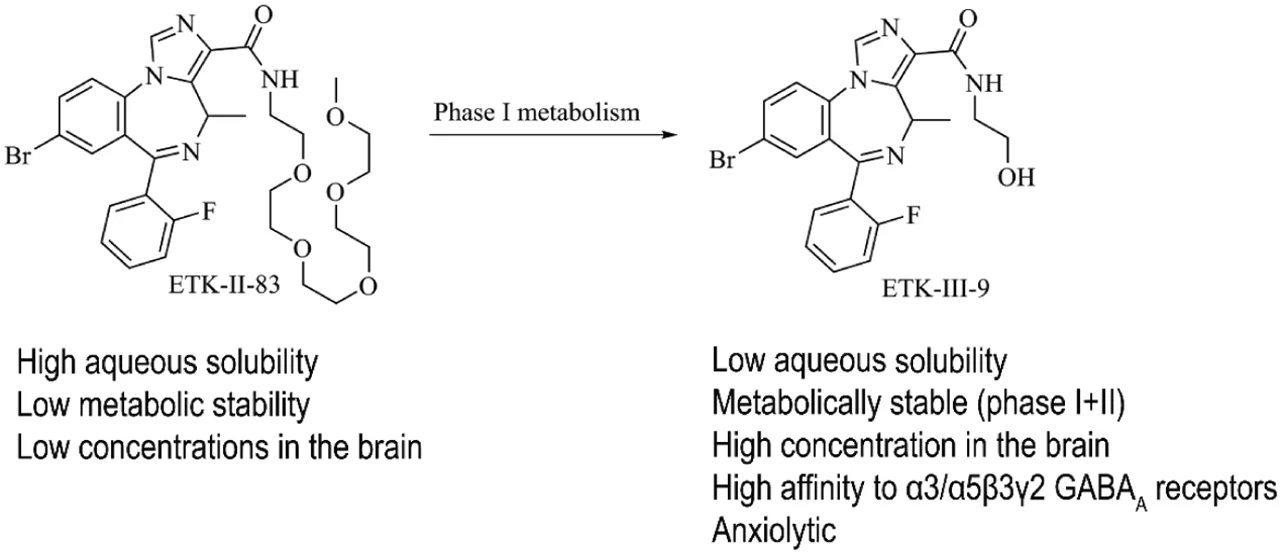

## 1 Introduction

Anxiety disorders are the most prevalent class of psychiatric disorders, with a lifetime prevalence in the United States of around 32% according to the National Comorbidity Survey Replication. Anxiety disorders arise from disruptions among the highly interconnected circuits of the brain in which the GABAergic system and its receptors have been implicated in the pathophysiology of anxiety and neurodegenerative disorders.^1^ First-line pharmacotherapy relies on benzodiazepines, which potentiate GABA-mediated inhibition, and on selective serotonin-reuptake inhibitors that modulate serotonergic tone. However, these medications are suboptimal in terms of efficiency and tolerability which necessitates the need for improved novel pharmacological agents. Benzodiazepines elicit their effects by enhancing the action of the inhibitory neurotransmitter gamma-aminobutyric acid (GABA) (positive allosteric modulation), thereby reducing neuronal excitability and inducing acute anxiolytic effects in anxiety patients.^2^ GABA_A_ receptors (GABA_A_R) belong to the class of ligand-gated chloride ion channels. To date, a large number of GABA_A_R subunits have been identified: α1–6, β1–3, γ1–3, δ, ρ1–3, θ, and π.^3^ Targeting specific GABA_A_R subtypes offers a novel pharmacological approach to dissociate therapeutic anxiolysis from undesirable side effects such as sedation. The imidazobenzodiazepine (IBZD) chemotype builds on the classic benzodiazepine scaffold but introduces a fused imidazole ring, enabling fine-tuned interactions with the benzodiazepine-binding site and better pharmacokinetic and pharmacodynamic profiles. We reported the anxiolytic effects of several IBZDs including HZ166 and KRM-II-81.^4^ These and other IBZDs selectively bind GABA_A_Rs containing the α2, α3, or α5 subunits with varying affinity toward the α1-containing GABA_A_Rs.^5^ It has been reported that α5-containing GABA_A_Rs are likely to mediate anxiolytic-like actions,^6^ whereas ligands with strong affinity toward α1-containing GABA_A_Rs induce sedation.^7^ Our group recently used pegylation to increase the solubility of IBZDs to enable aqueous formulations for nebulization.^8^ Further studies revealed that the *in vivo* half-lives of these pegylated IBZDs are sufficient to elicit a pharmacological response in the lung, but compound blood levels were very low indicating fast systemic metabolism.^9^ In the present study, we further explore this observation by generating a pegylated pro-drug ETK-II-83 and its structurally related metabolic product ETK-III-9 (Figure 1). We provide detailed preclinical characterization that includes physicochemical properties (solubility and permeability), *in vitro* characterization (cytotoxicity and GABA_A_R receptor binding), and *in vivo* anxiolytic effects (behavioral studies). Furthermore, we present the kinetics of metabolism *in vitro* (microsomal stability assays) and *in vivo* (pharmacokinetics). The significance of this study is that a pegylated IBZD in contrast to nonpegylated IBZDs can be formulated as an aqueous solution at high concentrations enabling intravenous, intramuscular, or as reported nebulized administration without significantly reducing the pharmacological effects of IBZDs.

**Figure 1:**
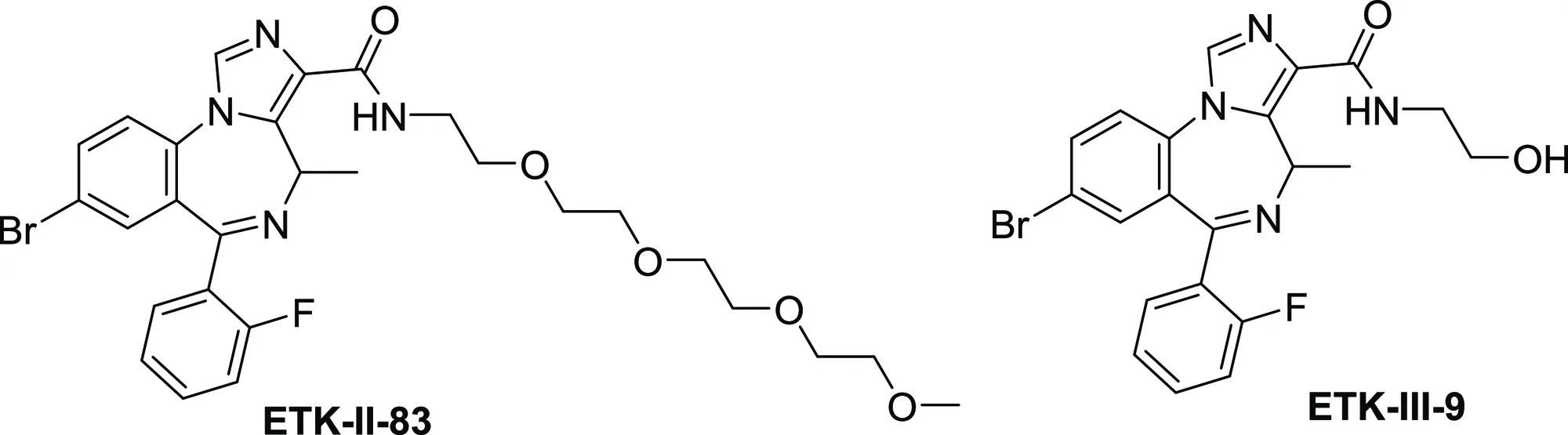
Structures of ETK-II-83 (pegylated prodrug) and its metabolite ETK-III-9.

## 2 Results and Discussion

ETK-II-83 and ETK-III-9 were synthesized starting with racemic MIDD0301, a strong bronchodilator that is currently being developed as an oral and nebulized therapeutic for asthma.^10,11^ The acid was treated with the coupling reagent HBTU (hexafluorophosphate benzotriazole tetramethyl uronium) in the presence of *N*-methylmorpholine (Figure 2). After 30 min at room temperature, the corresponding amine was added and the reaction was stirred for 90 min. The yields for ETK-II-83 and ETK-III-9 were 70.8% and 67.5%, respectively.

**Figure 2:**
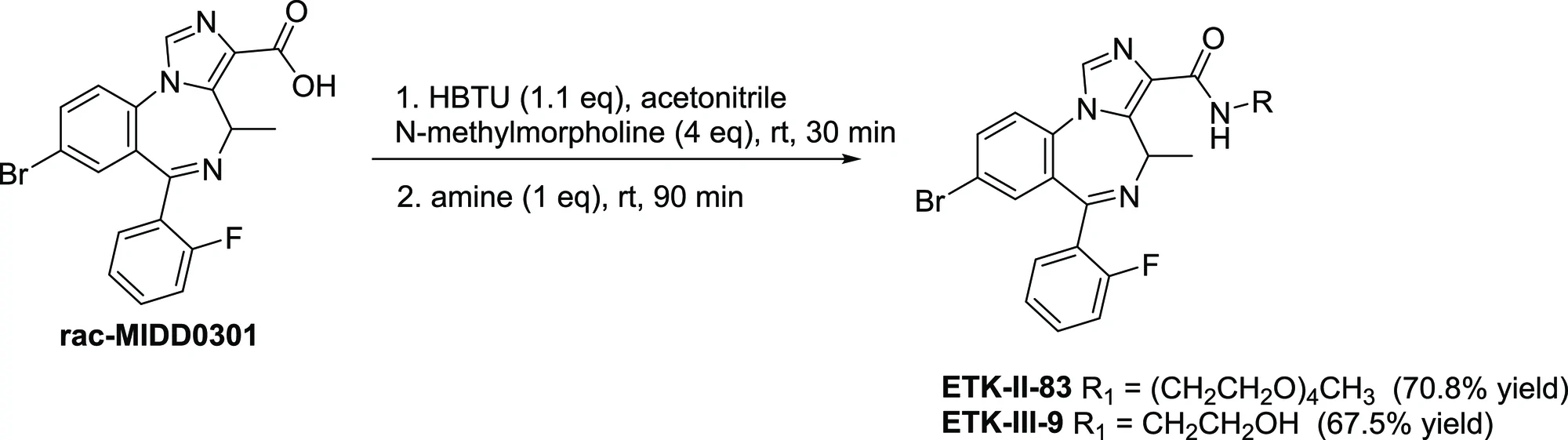
Synthesis of ETK-II-83 and ETK-III-9.

Next, we evaluated the aqueous solubility, permeability, cytotoxicity, and receptor binding of ETK-II-83 and ETK-III-9 (Table 1).

**Table 1:** Summary of Physiochemical Properties and Receptor Binding of ETK-II-83 and ETK-III-9

Physiochemical Properties	ETK-II-83	ETK-III-9
Aqueous solubility (μM)^a^	1121.6 ± 26.6	7.26 ± 1.87
Permeability (P_e_ (10^–5^ cm/s))^b^	1.77 ± 0.54	0.69 ± 0.05
Cell viability LD_50_ (μM)^c^	>300	>300
Binding to rat brain GABA_A_Rs (^3^H-flunitrazepam, IC50 (nM))^d^	206.4 ± 93.0	97.8 ± 39.1
Binding to KOR (^3^H-U69593, IC50 (nM))^e^	>10000	1590 ± 710
Binding to rat α1β3γ2 GABA_A_R (^3^H-flunitrazepam, % inhibition at 100 nM)^f^	39.5 ± 24.4	57.8 ± 18.1
Binding to rat α2β3γ2 GABA_A_R (^3^H-flunitrazepam, % inhibition at 100 nM)^f^	42.8 ± 9.3	65.7 ± 2.3
Binding to rat α3β3γ2 GABA_A_R (^3^H-flunitrazepam, % inhibition at 100 nM)^f^	49.5 ± 23.4	91.7 ± 6.7
Binding to rat α5β3γ2 GABA_A_R (^3^H-flunitrazepam, % inhibition at 100 nM)^f^	94.4 ± 16.7	110.1 ± 12.6

a Shake flask, water pH 7.4, mean ± SD.

b Permeability was measured using the parallel artificial membrane permeation assay at pH 7.4. Reference standards (P_e_) (10^–5^ cm/s): ranitidine (0.01), low permeability; naproxen (1.0), medium permeability; and verapamil (10.0), high permeability; mean ± SD.

c Cell viability was determined using HEK293 cells and CellTiter-Glo.

d Radioligand competition assay with rat brain extract (^3^H-flunitrazepam), dose–response analysis with nonlinear regression, IC50 ± 95% confidence interval.

e Radioligand competition assay with HEK293-expressing KOR using ^3^H-U69593, dose–response analysis with nonlinear regression, IC50 ± 95% confidence interval.

f Radioligand competition assay with cell membranes from GABA_A_R-expressing HEK293 cells (^3^H-flunitrazepam). Dpm (disintegrations per minute) values were normalized with mean dpm values for DMSO and mean dpm values in the presence of diazepam (50 μM), mean ± SD.

As expected, ETK-II-83 has a high aqueous solubility at pH 7.2 reaching a concentration of 1121 μM or 0.675 g/L. In contrast, metabolite ETK-III-9 is only sparsely soluble in water at pH 7.2 with a maximum concentration of 7.26 μM or 0.0033 g/L. In comparison, the structurally related compound midazolam has a solubility of less than 0.1 g/L at neutral pH.^12^ To overcome the limited solubility of midazolam and enable an aqueous formulation for intravenous and intramuscular administration, a low pH (<4) formulation has been developed taking advantage of the ring-opening reaction of 1,4-benzodiazepines.^13^ However, the IV solution can only be injected into large veins due to the pH difference and has to be administered slowly to avoid respiratory arrest. In contrast, aqueous solutions of ETK-II-83 can be generated at neutral pH. The permeabilities of ETK-II-83 and ETK-III-9 are comparable to naproxen measured by a parallel artificial membrane permeation assay. Based on this data, it is very likely that both compounds are orally available. No cellular toxicity was observed up to a concentration of 150 μM for embryonic kidney cells. A 25% reduction in viability was observed at 300 μM (see Supporting Information). Both compounds were screened with a panel of 45 CNS receptors for target engagement.^14^ Among these, binding for ETK-II-83 was only observed for the rat brain benzodiazepine site with an IC_50_ of 206.4 nM (Table 1). ETK-III-9 also displaced radioligand ^3^H-flunitrazepam with an IC_50_ of 97.8 nM and additionally showed weak affinity for the kappa opioid receptor (IC_50_ = 1.59 μM). Because of the abundance of GABA_A_R subtypes in the brain, a cell-based binding assay with specifically expressed GABA_A_R subtypes was carried out with 100 nM of ETK-II-83 and ETK-III-9 (Table 1). Displacement of ^3^H-flunitrazepam by ETK-II-83 and ETK-III-9 was observed for α1β3γ2, α2β3γ2, α3β3γ2, and α5β3γ2 GABA_A_R subtypes. These data were normalized to the negative control (DMSO) and positive control (diazepam) at 50 μM. The strongest displacement for ETK-II-83 was observed for the α5β3γ2 GABA_A_R, whereas ETK-III-9 interacted strongly with the α3β3γ2 and α5β3γ2 GABA_A_R subtypes. The subtype selectivity of ETK-II-83 and ETK-III-9 is similar to that of structurally related compounds.^15^ The concentration-dependent inhibition study showed that ETK-II-83 has an affinity of 18.3 nM for the α5β3γ2 GABA_A_R (Figure 3A). The IC_50_ for ETK-III-9 for this subtype was 7.6 nM. We observed that ETK-III-9 displaced more ^3^H-flunitrazepam than ETK-II-83 at a concentration of 1 μM. This prompted the analysis of additional GABA_A_R ligands including Ro 15-1788, diazepam, and clonazepam at a concentration of 50 μM (Figure 3B).

**Figure 3:**
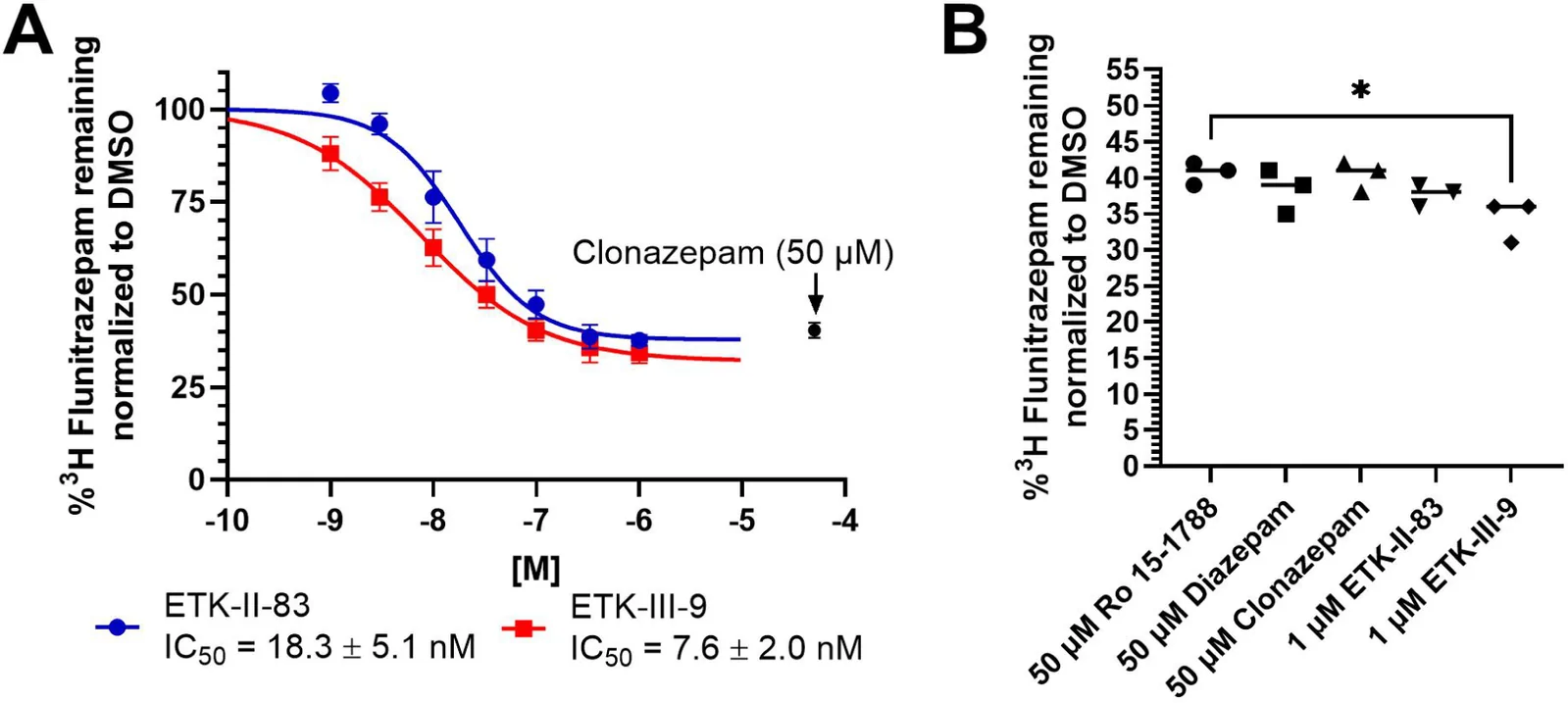
Radioligand (^3^H-flunitrazepam) competition assay with cell membranes from α5β3γ2 GABA_A_R-expressing HEK293 cells in the presence of compounds. Dpm values were normalized to the mean dpm values for DMSO. A) Concentration–response analysis with nonlinear regression, IC_50_ ± 95% confidence interval. Clonazepam was evaluated at a single concentration of 50 μM, mean ± SD; B) dpm values were normalized to mean dpm values for DMSO, *n* = 3, *p < 0.05 significance was calculated with 1-way ANOVA using Tukey’s multiple comparison test.

Only Ro 15-1788 at 50 μM showed significantly less displacement of ^3^H-flunitrazepam than ETK-III-9 at 1 μM. It can be hypothesized that flunitrazepam and ETK-III-9 bind an additional allosteric site on the α5β3γ2 GABA_A_R that is not accessible to Ro 15-1788 at a concentration of 50 μM. Other allosteric GABA_A_R binding sites such as the β2+/*γ2-* subunit interface have been observed to bind GABA_A_R ligands.^16^

Next, we determined the metabolic stability and free brain-to-blood ratios of ETK-II-83 and ETK-III-9 and summarized the results in Table 2.

**Table 2:** In Vitro Stability and Protein Binding of ETK-II-83 and ETK-III-9

In Vitro ADME	ETK-II-83	ETK-III-9
Phase I stability mouse liver microsomes (% remaining after 2h^a^	2.6 ± 1.0	88.2 ± 3.8
Phase II stability mouse liver microsomes (% remaining after 2h (glucuronidation)^b^	n.d.	96.1 ± 2.0
Ratio of bound and unbound compound in mouse plasma^c^	0.064 ± 0.011	0.097 ± 0.023
Ratio of unbound to bound compoundin mouse brain (fu)^d^	0.127 ± 0.021	0.066 ± 0.009

a Compounds at 10 μM were incubated with mouse liver microsomes and NADPH Regenerating System Solution A&B and quantified by LC–MS/MS after 2 h (*n* = 4), mean ± SD.

b Compounds at 10 μM were incubated with mouse liver S9 and UDP-glucuronic acid and quantified by LC–MS/MS after 2 h (*n* = 4), mean ± SD.

c Compounds at 10 μM were incubated with mouse plasma using microdialysis and quantified by LC–MS/MS after 4 h (*n* = 6), mean ± SD.

d Compounds at 10 μM were incubated with mouse brain homogenate using microdialysis and quantified by LC–MS/MS after 4 h (*n* = 6), mean ± SD.

We found that ETK-II-83 underwent rapid phase I metabolism. After 2 h, only 2.6% of ETK-II-83 was detected by LC–MS/MS (Table 2). The investigation of the microsomal stability reaction mixture led to the discovery of ETK-III-9 as one of the major metabolic products. A time-dependent experiment showed that the metabolism of ETK-II-83 *in vitro* occurred with a k = 0.130 min^–1^ and a half-life of 5.33 min (Figure 4A). In the same experiment, we quantified the formation of ETK-III-9 with a k = 0.036 min^–1^. The lower rate for the formation of ETK-III-9 supports the involvement of intermediates for this transformation, which included oxidation to a hemiacetal and subsequent hydrolysis to a PEG alcohol. To rule out other enzymatic degradations, we performed a stability study in mouse plasma with ETK-II-83 over a period of 120 min that did not lead to any degree of conversion (data not shown). Metabolic oxidation of ETK-II-83 occurred at the O–CH_2_ and O–CH_3_ carbons of the PEG chain. The metabolite ETK-III-9, without the additional ethylene glycol units, is very stable under these conditions with 88.2% remaining after 120 min (Table 2). The mass spectrum of the metabolic reaction of ETK-II-83 showed four major metabolites and their sodium adducts (Figure 4B). ETK-III-9 was observed as the major metabolite followed by metabolites with increasing PEG chain lengths (Figure 4D). The mass chromatograms can be found in the Supporting Information. To investigate if the metabolic breakdown is unique to ETK-II-83, we studied similar compounds with shorter and longer PEG chains (Figure 4C).^9^ We found that all these compounds formed ETK-III-9 within 120 min (Figure 4D).

**Figure 4:**
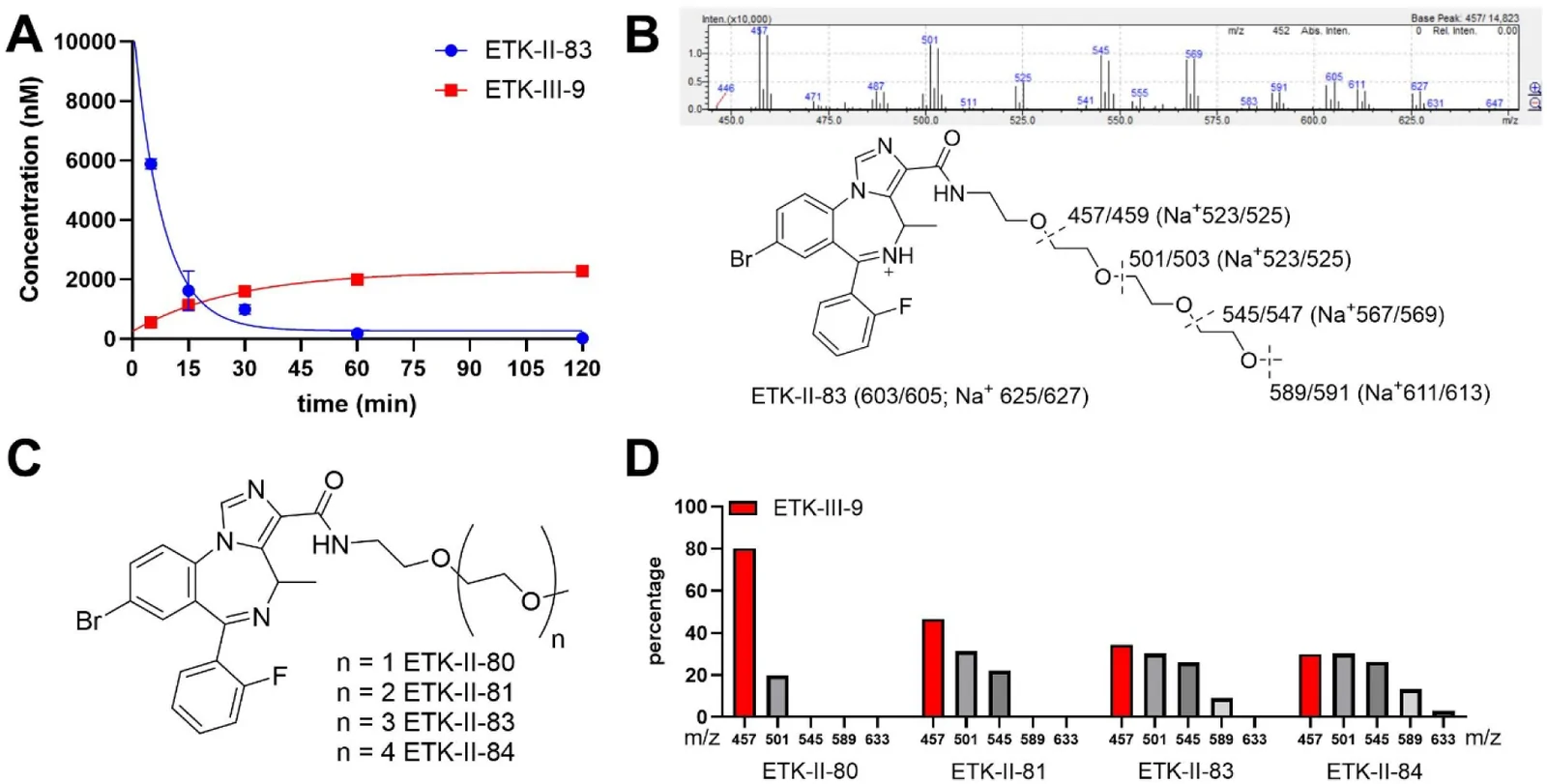
Metabolic stability studies (phase 1). A) time-dependent enzymatic conversion in the presence of NADPH and mouse liver microsomes monitoring the conversion of ETK-II-83 into ETK-III-9. Data are expressed as mean ± SD with *n* = 3 for each time point. Data were analyzed by nonlinear regression (one-phase decay) to calculate k and half-life; B) mass spectrum of microsomal assay with ETK-II-83 and structural identification of metabolites; C) structures of ETK-II-83-related compounds with longer and shorter PEG chains; D) percentages of each metabolite formed by microsomal oxidation of ETK-II-83 and its analogs.

We further analyzed the free blood and free brain ratios to enable the determination of total and free compound concentrations by using our pharmacokinetic data (Table 2). For this study, we used a rapid equilibrium dialysis device with chamber pairs divided by a membrane. Compounds incubated with mouse plasma or mouse brain homogenate were equilibrated for 4 h with a phosphate-buffered saline-filled chamber. Compound concentrations were quantified by LC–MS/MS, enabling the calculation of free plasma and free brain ratios. For ETK-II-83, we determined a free plasma ratio of 0.064 (6.4%) and a free brain ratio of 0.127 (12.7%). The free ratios for ETK-III-9 for plasma and brain were 0.097 (9.7%) and 0.066 (6.6%), respectively.

Next, we investigated the pharmacokinetics (PK) and metabolism in vivo. Therefore, two separate PK experiments were conducted. One with 40 mg/kg ETK-II-83 and the other with 40 mg/kg ETK-III-9 were used for oral administration in mice. The results are summarized in Figure 5.

**Figure 5:**
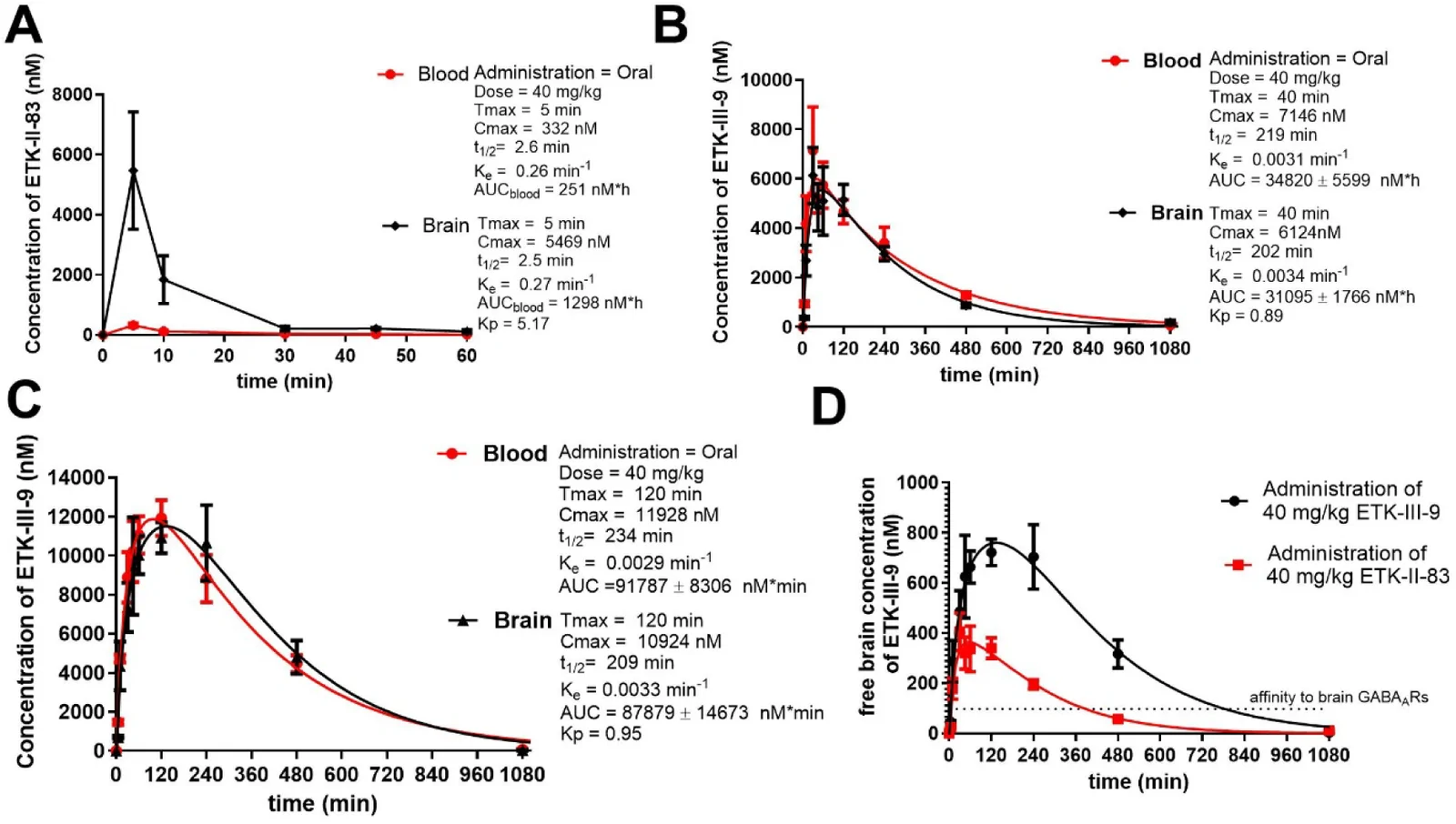
Pharmacokinetic analysis. A) oral administration of 40 mg/kg of ETK-II-83 to Swiss webster mice followed by quantification of ETK-II-83 in blood and brain; B) quantification of plasma and brain concentration of ETK-III-9 after oral administration of 40 mg/kg of ETK-II-83; C) oral administration of 40 mg/kg of ETK-III-9 to Swiss webster mice followed by quantification of ETK-III-9 in blood and brain; D) quantification of free brain concentration of ETK-III-9 after oral administration of 40 mg/kg of ETK-II-83 or ETK-III-9. Data are given as mean ± SEM and analyzed by nonlinear regression using a two-compartment model (biexponential equation for absorption and elimination).

Blood concentrations for ETK-II-83 peaked at 5 min for blood and brain samples and sharply declined over time with less than 50 nM remaining at 30 min in blood and brain (Figure 5A). Rapid metabolism could be the cause. Indeed, we showed that ETK-II-83 undergoes rapid phase I metabolism to ETK-III-9, which prompted us to quantify ETK-III-9 in parallel. Interestingly, high concentrations of ETK-III-9 were found in the blood and brain samples of ETK-II-83-treated animals (Figure 5B). Comparing blood and brain AUCs of ETK-II-83 and ETK-III-9 for animals treated with 40 mg/kg ETK-II-83, we observed a ratio of 1:138 for blood but only a 1:24 ratio for the brain. Thus, ETK-II-83 is absorbed very quickly and crosses the blood–brain barrier. Slower metabolism in the brain in comparison with systemic metabolism resulted in higher concentrations in the brain than in the blood. We have shown that ETK-III-9 is stable toward phase I and II metabolism (Table 2). This stability is reflected in the half-life of 219 min in blood and 202 min in the brain (Figure 5B). The brain-to-plasma partition coefficient of 0.89 (K_P_) is similar to K_P_ values reported for structurally related compounds.^15^ The PK study with ETK-III-9 showed efficient and slow absorption with increasing concentrations for 2 h (Figure 5C). The elimination half-lives (blood and brain) for ETK-III-9 in this experiment matched the half-lives reported for ETK-III-9 when animals were treated with ETK-II-83. Also, the calculated K_P_ value of 0.95 is similar. The AUCs found for the brain and blood are significantly higher for the administration of ETK-III-9 (about 3-fold) than for the administration of ETK-II-83 at the same dose. One possible explanation could be the degradation of ETK-II-83 in the mouse intestine, preventing complete absorption. Using the determined free brain fraction of ETK-III-9, we calculated the free concentrations of ETK-III-9 in the mouse brain after the administration of ETK-II-83 and ETK-III-9 (Figure 5D). We found that for several hours, the free brain concentrations of ETK-II-83 and ETK-III-9 are higher than their IC50 values for brain-expressed GABA_A_Rs.

The open field test (OFT) is a widely used preclinical model for assessing the exploratory locomotion of rodents. It has been reported that positive allosteric GABA_A_R modulators, especially those with strong binding to the α1 subunit-containing GABA_A_Rs, induce sedation and reduce locomotion.^17^ Here, we investigated the effect of orally administered ETK-II-83 and ETK-III-9 in comparison with the nonsubtype-selective positive allosteric GABA_A_R modulator diazepam (Figure 6).

**Figure 6:**
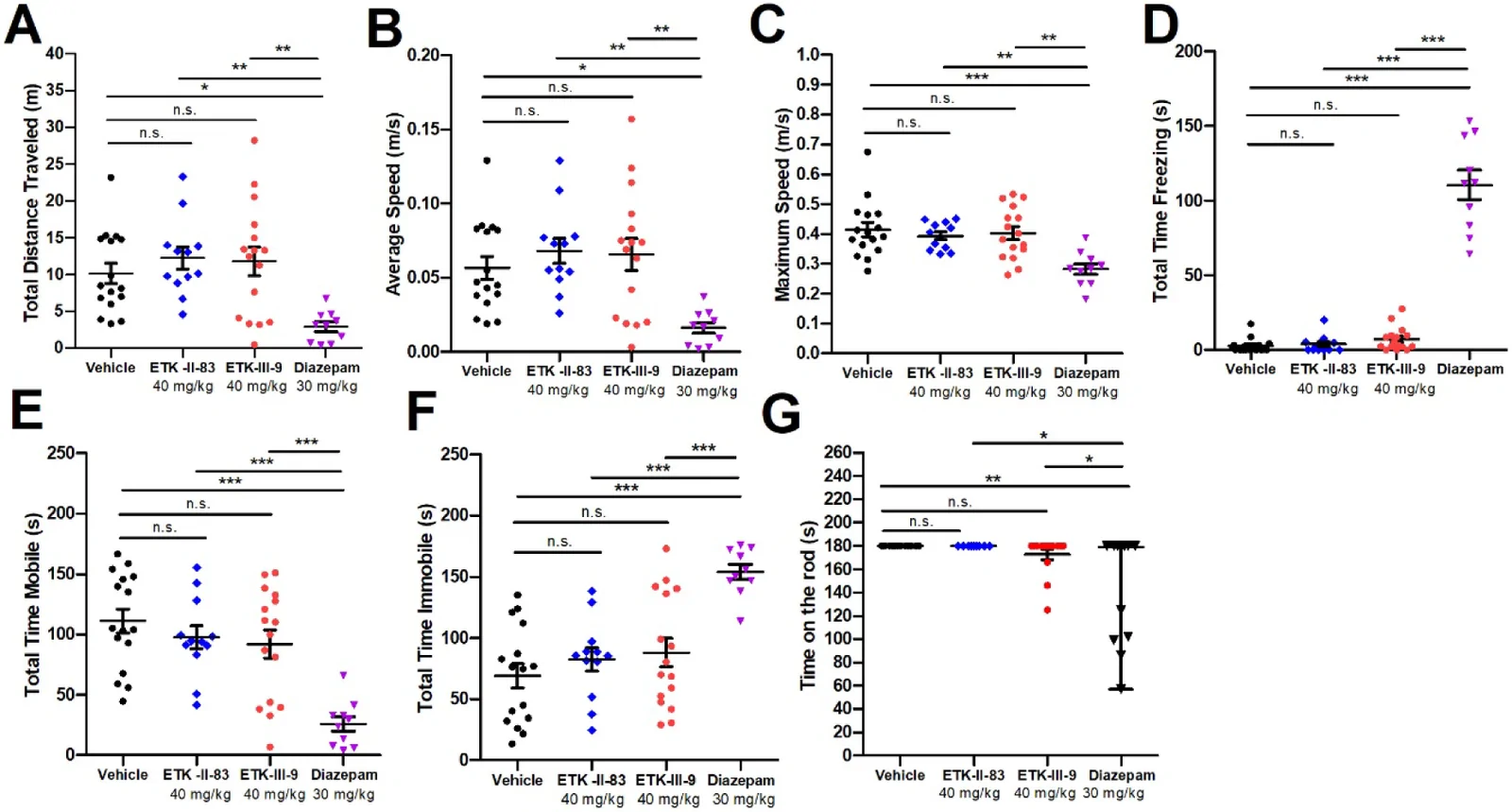
Open field and rotarod test (mice). Compounds were administered orally and testing was performed 1 h after administration. For the open field test, locomotion was recorded for 3 min in an open-field acrylic arena (40 × 40 × 40 cm). A) total distances traveled (m); B) average speed (m/s); C) maximum speed (m/s); D) total time freezing (s); E) total time mobile (s); F) total time immobile (s); G) for the rotarod test, mice were monitored on a rotarod apparatus for 3 min 60 min after oral administration. The time (s) of fall was recorded if it occurred before the end of the experiment. Data are expressed as scatter plots, means, and SEM (*n* = 16). N.S. *p* > 0.5, *p < 0.05, **p < 0.01, and ***p < 0.001 significances were calculated with 1-way ANOVA using Tukey’s multiple comparison test.

We observed that ETK-II-83 and ETK-III-9 did not influence the overall behavior of mice at the dose of 40 mg/kg. In comparison to the vehicle-treated mice, we did not observe any changes in total distance traveled (Figure 6A), average speed (Figure 6B), maximum speed (Figure 6C), total time freezing (Figure 6D), total time mobile (Figure 6E), and total time immobile (Figure 6F). Diazepam at a lower dose of 30 mg/kg induced a significant reduction in mobility. Mice moved 7.2 m less than vehicle-treated mice with an average speed reduction of 70%. Also, diazepam-treated mice often stopped staying immobile and did not move at all (freezing). Similar effects for diazepam doses up to 2 mg/kg using C57BL/6J mice have been reported.^18^ Importantly, ETK-II-83 and ETK-III-9 did not induce such a behavioral change even at a higher dose than diazepam. This could be related to weaker binding to the α1β3γ2 GABA_A_R (Table 1) in contrast to diazepam that binds these subtypes with almost equal affinity.^15^ We also determined the ability of treated mice to complete balancing on a rotating rod for 3 min (rotarod test) (Figure 6G). Here, we observed that all ETK-II-83-treated mice completed this task. Three out of ten ETK-III-9-treated mice did not complete this test. This rate was higher for diazepam-treated mice, resulting in five animals that did not complete this test indicating some degree of sensorimotor inhibition.

Next, we evaluated ETK-II-83 and ETK-III-9 with an elevated plus maze paradigm, which was widely used for screening new potential antianxiety drug candidates (Figure 7).^19^

**Figure 7:**
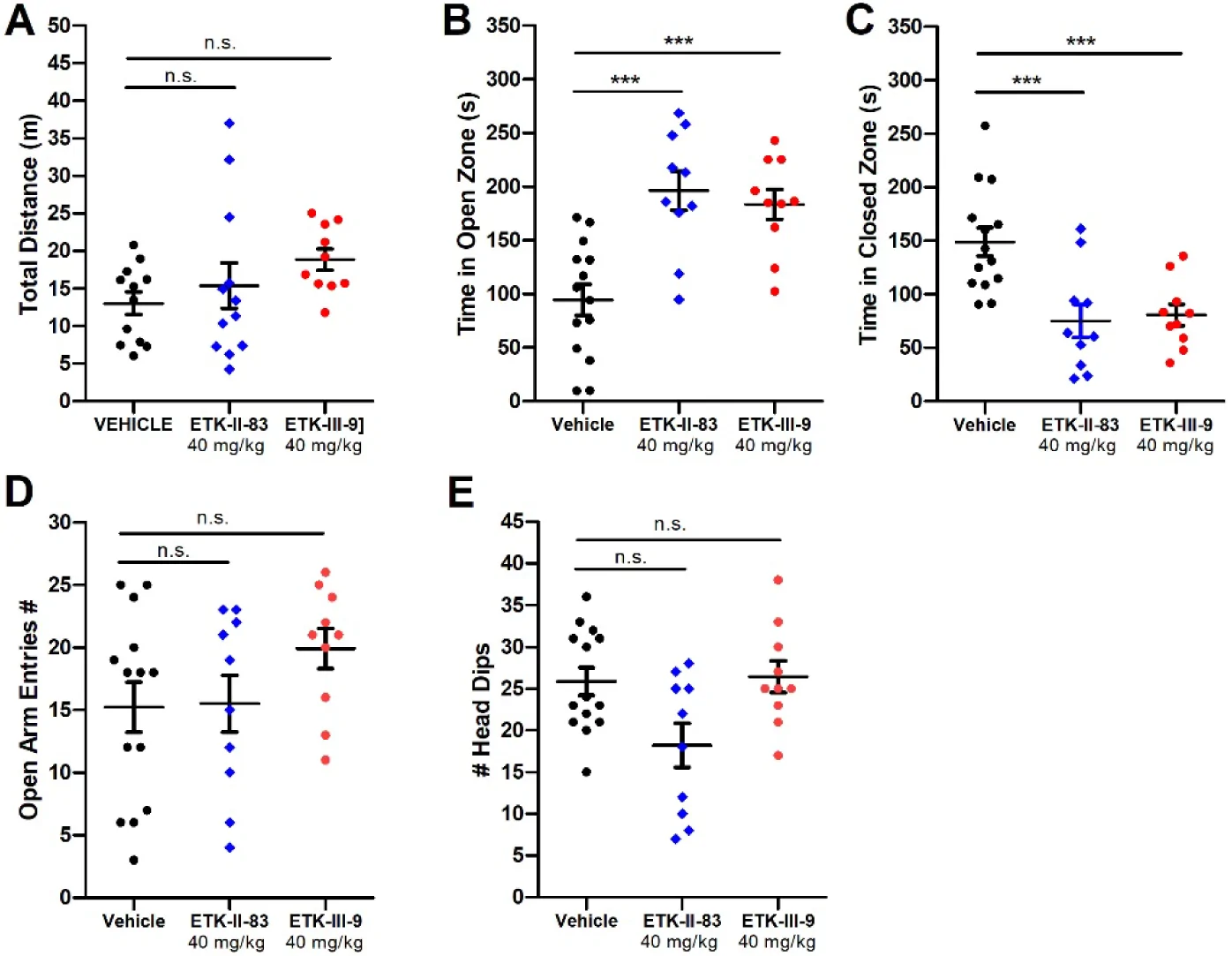
Elevated plus maze test. ETK-II-83 and ETK-III-9 were administered orally 60 min before mice were monitored for 5 min in an elevated plus maze. A) total distance (m); B) time in open zone (s); C) time in closed zone (s); D) number of open arm entries; E) number of head dips. Data are expressed in scatter plots, means, and SEM (*n* = 10). N.S. *p* > 0.05, and ***p < 0.001 significance were calculated with 1-way ANOVA using Tukey’s multiple comparison test.

We confirmed that ETK-II-83 and ETK-III-9 did not change the total distance traveled as we previously determined with the open field test (Figure 7A). However, we found that animals treated with ETK-II-83 and ETK-III-9 spent more time in the open zone, which is related to anxiolysis (Figure 7B). Inversely, these mice spent less time in the closed zone (Figure 7C). We also quantified open arm entries and head dips, which were not statistically different from vehicle control animals (Figure 7D and E). ETK-III-9 is structurally related to compounds like GL-II-73 and SH-053-R-CH3-2′F, which have been reported to be α5-selective positive GABA_A_R modulators.^15,20^ Administration of these ligands increased the time that mice spent in the open arm at a dose of 10 mg/kg.

The rodent marble-burying test is another measure used to study anxiety and compulsive-like behaviors and is often used to characterize the effects of novel anxiolytics.^21,22^ Here, we used the same dose and unconditioned mice to investigate the effects of ETK-II-83 and ETK-III-9 and summarized the results in Figure 8.

**Figure 8:**
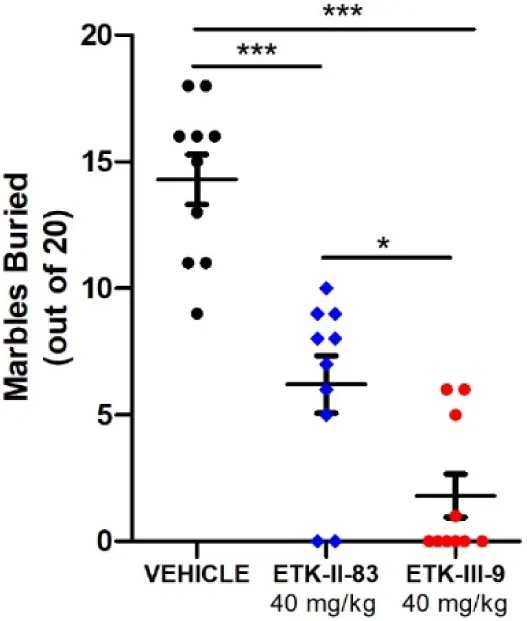
Marble burying test. Female Swiss webster mice were monitored in a standard polycarbonate rat cages (26 cm × 48 cm × 20 cm) with 5 rows of 4 marbles undisturbed for 30 min. Buried marbles were counted and depicted as scatter plots, mean, and SEM (*n* = 10). *p < 0.05 and ***p < 0.001 significance were calculated with 1-way ANOVA using Tukey’s multiple comparison test.

We observed that ETK-II-83 and ETK-III-9 had profound effects on the mouse behavior in this test. Using a dose of 40 mg/kg, which was previously reported to have no effect on the mobility of mice, we observed a significant reduction in buried marbles. The effect was even more pronounced for the mice that were treated with ETK-III-9 than for those treated with ETK-II-83.

In conclusion, while direct administration of ETK-III-9 provides superior systemic and brain exposure at equivalent doses, ETK-II-83 as a prodrug may offer a significantly faster attainment of therapeutic brain concentrations. Furthermore, ETK-II-83 has high aqueous solubility that will allow routes of administration such as intravenous and intramuscular that are not feasible with ETK-III-9. The optimal development strategy should consider whether rapid onset or sustained high exposure is more critical for efficacy and whether dose adjustments can compensate for the reduced bioavailability associated with prodrug metabolism.

## 3 Methods

Chemicals and solvents were purchased from commercial sources and used without further purification. Reaction progress was monitored by silica gel thin-layer chromatography with a fluorescence indicator (Macherey-Nagel Inc.). ^1^H, ^13^C, and ^19^F-NMR spectra were recorded with a Bruker 500 MHz instrument with the chemical shifts in δ (ppm) reported by reference to the deuterated solvents as an internal standard: CDCl_3_: δ = 7.20 ppm (^1^H NMR) and δ = 77.00 ppm (^13^C NMR). HRMS spectral data were recorded using an LCMS-QTOF spectrometer (Shimadzu). High-performance liquid chromatography (Shimadzu Nexera series HPLC) coupled with a Photo Diode Array detector (PDA, Shimadzu SPD-M30A) and a single quadrupole mass analyzer (LCMS-2020, Shimadzu, Kyoto, Japan) was used for purity analysis (absolute area %). Analytes were separated using a Restek Pinnacle-C18 (4.6 mm × 50 mm, 5 μm particle size) column with a gradient of water and methanol (0.1% formic acid) at a flow rate of 0.8 mL/min (see Supporting Information for analytical spectra).

### 3.1 Synthesis

Under a nitrogen atmosphere, racemic 8-bromo-6-(2-fluorophenyl)-4-methyl-4H-benzo­[f]­imidazo­[1,5-*a*]­[1,4]­diazepine-3-carboxylic acid^11^ (414 mg, 1.0 mmol) and HBTU (405 mg, 1.07 mmol) were added to the flask, followed by anhydrous acetonitrile (10 mL) and *N*-methylmorpholine (425 μL, 3.86 mmol). The solution was allowed to react at room temperature for 30 min followed by the addition of 2,5,8,11-tetraoxatridecan-13-amine (269 mg, 1.3 mmol). The solution was allowed to stir at room temperature for 90 min. The reaction mixture was concentrated under reduced pressure before redissolving in CHCl_3_ (10 mL). The organic layer was washed with saturated aqueous NH_4_Cl (1 × 10 mL). The aqueous layer was extracted (3×) with CHCl_3_ (10 mL each). The organic layers were combined and washed with brine (1× 25 mL). The organic layer was dried over MgSO_4_, filtered and concentrated under reduced pressure to yield a pale-yellow residue. The residue was loaded onto a precolumn with CHCl_3_ and separated by automated column chromatography (Biotage): 0–0% MeOH:CHCl_3_ (4 CVs), then 0–8% MeOH:CHCl_3_ (30 CVs). ETK-II-83 was obtained as a pale-white-yellow thick residue with a yield of 427 mg (70.8%): ^1^H NMR (500 MHz, CDCl_3_) δ 7.82 (s, 1H), 7.69 (d, J = 7.7 Hz, 1H), 7.61 (t, J = 7.2 Hz, 1H), 7.54 (s, 1H), 7.44 (t, J = 4.2 Hz, 1H), 7.40 (s, 1H), 7.24 (dt, J = 0.9, 7.5 Hz, 1H), 7.01 (t, J = 9.3 Hz, 1H), 6.87 (q, J = 7.2 Hz, 1H), 3.69–3.53 (m, 16H), 3.36 (s, 3H), 1.26 (d, J = 7.3 Hz, 3H); ^13^C NMR (126 MHz, CDCl_3_) δ 162.72 (s, 1C), 162.29 (s, 1C), 161.08 (s, 1C), 159.09 (s, 1C), 138.83 (s, 1C), 134.64 (s, 1C), 133.85 (s, 1C), 133.45 (s, 1C), 132.96 (s, 1C), 131.96 (d, J = 8.3 Hz, 1C), 131.70 (s, 1C), 131.30 (d, J = 8.9 Hz, 1C), 128.58 (d, J = 12.3 Hz, 1C), 124.51 (d, J = 2.9 Hz, 1C), 123.54 (s, 1C), 120.68 (s, 1C), 116.10 (d, J = 21.5 Hz, 1C), 71.93 (s, 1C), 70.62 (s, 1C), 70.61 (s, 1C), 70.53 (s, 1C), 70.50 (s, 1C), 70.39 (s, 1C), 70.09 (s, 1C), 59.01 (s, 1C), 49.91 (s, 1C), 38.66 (s, 1C), 15.03 (s, 1C).; ^19^F NMR (471 MHz, CDCl_3_) δ −112.31 (s, 1F).; HRMS (ESI/Q-TOF): *m*/*z* [M + H]^+^ pred. for C_28_H_32_O_5_BrFN_4_: 603.16129; meas.: 603.16129; HPLC Purity: 91.6%. For ETK-III-9, the same protocol was followed but using ethanolamine (117 μL, 1.94 mmol) as the amine. ETK-III-9 was obtained as a pale-white-yellow thick residue that solidified upon standing. 308 mg (67.5%): ^1^H NMR (500 MHz, CDCl_3_) δ 7.84 (s, 1H), 7.72 (d, J = 7.3 Hz, 1H), 7.65 (t, J = 7.2 Hz, 1H), 7.59 (s, 1H), 7.48 (q, J = 3.1 Hz, 1H), 7.45 (q, J = 2.4 Hz, 1H), 7.26 (q, J = 2.8 Hz, 1H), 7.04 (t, J = 9.3 Hz, 1H), 6.88 (q, J = 7.3 Hz, 1H), 3.82 (t, J = 4.9 Hz, 2H), 3.63 (m, J = 4.9 Hz, 1H), 3.56 (m, J = 4.9 Hz, 1H), 1.29 (d, J = 7.3 Hz, 3H); ^13^C NMR (126 MHz, CDCl_3_) δ 164.10 (s, 1C), 162.47 (s, 1C) 159.12 (s, 1C), 156.75 (s, 1C), 139.11 (s, 1C), 134.72 (s, 1C), 133.06 (s, 1C), 131.39 (s, 1C), 131.29 (s, 1C), 124.56 (s, 1C), 123.51 (s, 1C), 120.92 (s, 1C), 116.23 (s, 1C), 116.06 (s, 1C), 63.13 (s, 1C), 49.86 (s, 1C), 42.62 (s, 1C), 14.96 (s, 1C); ^19^F NMR (471 MHz, CDCl_3_) δ −112.07 (d, J = 86.4 Hz, 1F); HRMS (ESI/Q-TOF): *m*/*z* [M + H]^+^ pred. for C_21_H_18_O_2_BrFN_4_: 457.06699; meas.: 457.06789; HPLC Purity: 95.08%.

### 3.2 Aqueous Solubility

Three milligrams of each compound was added to 500 μL of PBS buffer at pH 7.4. The solutions were vortexed for 10 s, sonicated for 2 min, and agitated with a horizontal shaker in a closed vial for 24 h. The mixtures were transferred to a 2 mL Eppendorf tube and centrifuged for 5 min at 16,000 × *g* followed by filtration through a 0.22 μm cellulose acetate Spin-X centrifuge filter (Costar). 200 μL of filtrate was transferred to a new 2 mL Eppendorf tube, and 200 μL of methanol was added and then subsequent dilution was made in 50:50 methanol/PBS buffer water if necessary. After mixing, 50 μL were transferred into a 384-well plate (Corning UV Star, 781801) for UV detection at 250–600 nm (Tecan M1000). The absorbance of the corresponding 50:50 methanol/PBS blank solution was recorded and subtracted from the calibration and compound absorbance curves. The concentration of each solution was determined with a calibration curve in 50:50 methanol/PBS buffer. The assay was carried out with three independent samples of each compound. The concentration of each compound is given as mean ± SD.

### 3.3 Permeability

The artificial membrane was prepared by carefully pipetting 15 μL of the 5% (v/v) hexadecane in hexane solution into each of the wells of the donor plate. The plate was placed in a fume hood for 1 h to ensure complete evaporation of the hexane. After the hexane had evaporated, 300 μL of PBS with 5% (v/v) DMSO was added to each of the wells of the acceptor plate. The hexadecane-treated donor plate was then placed on top of the acceptor plate taking care that the underside of the membrane was completely in contact with the solution in each of the acceptor wells. 300 μM solutions were prepared for each compound in 5% (v/v) DMSO in PBS and 150 μM was transferred in triplicate to the donor wells. A lid was placed on the plate, and the entire plate sandwich was placed in a closed humid environment and agitated at 100 rpm for 18 h. The donor plate was removed and 50 μL of the acceptor solution was transferred to the UV plate. Calibration curves were generated from a 100 μM solution in 5% (v/v) DMSO in PBS. The absorbance of the solutions in the UV plate was then scanned from 250 to 600 nm to identify the most suitable wavelength with an absorbance between 0.5 and 1. The absorbance of the corresponding DMSO PBS blank was recorded and subtracted from the absorbance of equilibrium and test solutions.

1 $$Pe=\left\{C\times -\ln\left(1-\frac{{[Drug]}_{A}}{{[Drug]}_{E}}\right)\right\},,\mathrm{where}\;C=\left(\frac{V_{A}\times V_{D}}{\left(V_{D}+V_{A}\right)A\times T}\right)$$

The relative permeability (cm/s) of the small molecules was calculated with eq 1, where *V_D_* is the volume of the donor well in cm^3^ (150 μL), *V_A_* is the volume of the acceptor well in cm^3^ (300 μL), *A* is the active surface area of the membrane in cm^2^ (0.283 cm^2^), *T* is the incubation time of the assay in seconds, [*Drug*]_*A*_ is the absorbance of the compound in the acceptor well after the incubation period, and [*Drug*]_*E*_ is the absorbance of the compound at the concentration of the theoretical equilibrium (as if the donor and acceptor solutions were simply combined). The value for each compound was determined as mean ± SD.

### 3.4 Cell Viability

Human embryonic kidney (HEK293T) cells were added to sterile white, optical bottom 384-well plates at a density of 15,000 cells/well (20 μL). Plates were incubated for 4 h at 37 °C with 5% CO_2_ before two transfers of 100 nL of serially diluted (1:2 in DMSO) compounds (final maximum concentration of 300 μM) were transferred using a Tecan Freedom EVO liquid handling system with a 100H stainless steel pin tool. The controls for the cytotoxicity assay were 3-dibutylamino-1-(4-hexyl-phenyl)-propan-1-one (150 μM in DMSO, positive control) and DMSO (negative control). After 18 h, assay plates were evaluated by adding 15 μL of the CellTiter-Glo Luminescence Assay Kit (Promega, Madison, WI) to each well, followed by detection of luminescence with a Tecan M1000 plate reader. Controls were measured on each plate enabling data normalization. Two independent experiments were performed in quadruplicate, and data were analyzed using nonlinear regression with variable slope (GraphPad Prism). (The dose–response curve graph is available as Supporting Information.) Because less than 50% of the cells died at concentrations of 300 μM, it can be assumed that the LD_50_ of both compounds is higher than 300 μM.

### 3.5 Rat Brain Binding (GABA_A_Rs)^14^

Rat brain membranes were homogenized on ice in a buffer containing 50 mM Tris–HCl, pH 7.4, and a protease inhibitor using a hand-held homogenizer. The mixture was centrifuged at 1000 × *g* for 10 min at 4 °C to obtain the supernatant. The supernatant was then centrifuged at 40,000 × *g* for 20 min followed by the removal of the supernatant and the addition of ice-cold lysis buffer. The homogenization–centrifugation procedure was repeated three times. The final pellet was resuspended in the same buffer and homogenized followed by the addition of [^3^H]-flunitrazepam (4.0 nM) and serially diluted compound concentrations in DMSO. Clonazepam was evaluated in parallel to verify that the obtained IC50 for this assay was similar to previously obtained IC50 values for clonazepam. After 90 min, the reactions were vacuum-filtered onto 0.3% polyethylenimine-soaked filter followed by three washes with cold PBS buffer. Scintillation solution was added to microwave-dried filters on a hot plate. Radioactivity was measured using a MicroBeta counter. The data (*n* = 6 for each concentration) were analyzed by nonlinear regression with a variable slope (GraphPad Prism). The data were given as IC50 and 95% confidence intervals.

### 3.6 KOR Binding Assay^14^

Radioligand binding assays are carried out in a final volume of 125 μL per well in an appropriate binding buffer. The homogenate of HEK293 cells that overexpress KOR was incubated with [^3^H]-U69593 (0.83 nM) and serially diluted compounds in DMSO at room temperature and in the dark for 90 min. Reactions were stopped by vacuum filtration onto 0.3% polyethylenimine (PEI)-soaked 96-well filter mats using a 96-well Filtermate harvester, followed by three washes with cold PBS buffer. Scintillation cocktail was then melted onto the microwave-dried filters on a hot plate, and radioactivity was counted in a MicroBeta counter. The data (*n* = 6 for each concentration) were analyzed by nonlinear regression with variable slope (GraphPad Prism). The data were given as IC50 and 95% confidence interval.

### 3.7 Subtype-Specific GABA_A_R Competition Binding Assays

HEK-293 cells were transfected with cDNAs encoding rat GABA_A_ receptor subunits subcloned into pCI expression vectors using the JetOPTIMUS (Polyplus) transfection reagent. 2.5 million cells were seeded into a 10 cm cell culture dish containing 9 mL of culture medium 1 day prior to transfection. 5 μg of DNA at a ratio of α:β:γ=1:1:3 was diluted in 1 mL of JetOPTIMUS-buffer and 10 μL of reagent was added. After 10 min of incubation at room temperature, the mixture was pipetted onto the cells. Cells were harvested 72 h after transfection by scraping into phosphate-buffered saline. After centrifugation (10 min, 3000 × *g*, 4 °C), cells were resuspended in TC50 (50 mM Tris–citrate, pH = 7.1), homogenized with an Ultra-Turrax (IKA, Staufen, Germany), centrifuged (20 min, 5000 × *g*, 4 °C), and the pellet was frozen at −20 °C until needed. For the radioligand membrane displacement assays, frozen membranes were thawed, resuspended, and incubated for 90 min at 4 °C in a total of 400 μL of TC50/NaCl (50 mM Tris–citrate, pH = 7.1; 150 mM NaCl), 100 nM (Table 1) or increasing concentrations (Figure 3) of ETK-II-83 or ETK-III-9, and 2 nM ^3^H-flunitrazepam in the absence or presence of either 50 μM diazepam, clonazepam, or Ro 15–1788 (also called flumazenil). The final DMSO concentration was 0.5%. Membranes were filtered through Whatman GF/B filters and rinsed twice with 4 mL of ice-cold 50 mM Tris–citrate buffer. Filters were transferred to scintillation vials and subjected to liquid scintillation counting after the addition of 3 mL of Rotiszint Filter liquid scintillation cocktail (Carl Roth, Germany) using a TriCarb 4910TR from PerkinElmer. Dpm values for compounds in the presence of 50 μM diazepam were subtracted from dpm values in the absence of diazepam to correct for nonspecific binding of ^3^H-flunitrazepam for the experiments described in Table 1. In Figure 3A, no correction for nonspecific binding was made. Each percentage was based on one or more independent experiments performed in triplicate. A scatter plot is provided in the Supporting Information. For the concentration-dependent studies, ETK-II-83 and ETK-III-9 were added to resuspended membranes at concentrations ranging from 1 nM to 1 μM. The same membrane preparation was used for vehicle DMSO and other GABA_A_R ligands at 50 μM. Dpm values measured after following the protocol above were normalized to the mean dpm for DMSO-treated membranes. All experiments were conducted with *n* = 3. Normalized data for ETK-II-83 and ETK-III-9 were analyzed by nonlinear regression and reported as IC50 ± 95% confidence interval. Normalized data for GABA_A_R ligands were reported as mean ± SD. Significance was calculated with 1-way ANOVA using Tukey’s multiple comparison test.

### 3.8 Microsomal Stability Studies

For detailed protocols and data on phase I and II metabolism, see Supporting Information.

### 3.9 Mouse Brain and Plasma Binding

A rapid equilibrium dialysis device (Thermo Scientific) was used to determine the free concentrations in mouse blood and brain. Therefore, 495 μL of mouse blood was combined with 5 μL of a 1 mM solution of ETK-II-83 or ETK-III-9 to obtain a final concentration of 10 μM. For the free brain fraction, brain samples were homogenized with 4 equiv of PBS and 495 μL of this suspension was combined with 5 μL of a 1 mM solution of ETK-II-83 or ETK-III-9 to obtain a final concentration of 10 μM. 500 μL aliquot of each solution was transferred into the red-marked retainer well of a rapid equilibrium dialysis device. 500 μL of PBS buffer was added to the adjacent buffer chamber. This process was repeated three times at different locations on the plate to achieve *n* = 3 for this study. The plate was sealed with tape and placed into a shaker for 4 h using an orbital shaking rate of 250 rpm. A 50 μL sample from the red well was transferred to an Eppendorf tube and combined with 50 μL of PBS. Similarly, a 50 μL sample from the buffer well was transferred to an Eppendorf tube and combined with 50 μL of a mouse plasma sample or mouse brain sample. Each combined sample was quenched with 120 μL of cold methanol containing 10 μM of 4,5-diphenylimidazole as an internal standard. The mixtures were vortex-mixed, incubated on ice for 30 min, and centrifuged at 14,000 × *g* for 10 min. The supernatants were transferred to clean tubes and evaporated to dryness under a gentle stream of nitrogen. The dried residues were reconstituted in 150 μL of methanol, sonicated, and centrifuged. The samples were subsequently passed through Spin-X filters by centrifugation at 500 × *g* for 2 min, and 100 μL of the filtrate was transferred to autosampler vials for analysis using an LC–MS/MS system (Shimadzu LCMS-8040, Shimadzu, Japan). The ratio of the peak areas of the internal standard and the test compounds was calculated for each sample. The free compound ratios were determined by dividing the ratio of the buffer chamber by the ratio of the plasma or brain chamber multiplied by 100. The unbound fraction in diluted brain tissue for each compound was calculated using the appropriate dilution correction. Analysis was conducted with a Shimadzu Nexera X2 LC30AD (Shimadzu, Kyoto, Japan) using a 5 μL injection volume. Separation was achieved by reverse-phase chromatography using an Eclipse Plus II RRHD Extend-C18 column. A 2.1 mm × 50 mm column with a 1.8 μm particle size was used with a 0.4 mL/min flow rate. The mobile phase was methanol and water (both containing 0.1% formic acid). Program: 35% B (0 min) → 99% B (4 min), 99% B (4 min) → 99% B (2 min), hold at 99% B (2 min), return to 35% B (0.10 min), and hold at 35% B (1 min). The column temperature was at 25 °C. Analysis was performed with positive ionization using MRM (multiple reaction monitoring) with a Shimadzu 8040 (Shimadzu, Kyoto, Japan) using electrospray, as well as atmospheric pressure ionization (DUIS). The following transitions were monitored in MRM mode: Ion pairs for ETK-II-83 were *m*/*z* 603.10 > 439.00, *m*/*z* 603.10 > 396.00, *m*/*z* 603.10 > 421.95; ion pairs for ETK-III-9 were *m*/*z* 457.00 > 396.00, *m*/*z* 457.00 > 368.00, m/z 457.00 > 326.90; internal standard *m*/*z* 221.00 > 194.15, *m*/*z* 221.00 > 165.15, *m*/*z* 221.00 > 91.05. The collision energy was adjusted for each ion transition. The heating block temperature was set to 400 °C. The drying gas flow rate was 15 L/min, and the desolvation line temperature was 250 °C. The nebulizing gas flow rate was 1.5 L/min, and a voltage of 4.5 kV was used to the needle and interface. Data acquisition and processing were performed using LabSolutions software.

### 3.10 Animals

Eight- to 10-week-old Swiss Webster female mice [20–25 g] were purchased from Charles River Laboratories and were housed in a pathogen-free environment with a 12-h light/dark cycle, with mouse chow and water provided ad libitum. All procedures involving animals were performed under the auspices of the institutional guidelines as defined by the UWM Institutional Animal Care and Use Committee (approved IACUC numbers are 22-23 #30 and 23-24 #17). ETK-II-83 and ETK-III-9 were formulated by dissolving them in hot PEG400 (2.5% v/v), followed by the addition of a 2% hydroxypropylmethylcellulose solution (97.5% v/v), and administered to each mouse via oral gavage.

### 3.11 Pharmacokinetic Study

Mice were randomly assigned to groups of four per time point and given a single 100 μL dose of the formulated compound via oral gavage. Mice were then euthanized at 5, 10, 30, 45, 60, 120, 240, 480, and 1080 min time points, followed by blood collection (+heparin) and brain harvesting for bioanalysis. To 100 μL blood aliquots were added 300 μL of cold acetonitrile and vortexed and sonicated for 30 s followed by centrifugation at 7500 × *g* for 10 min. 300 μL of the supernatant was transferred to clean tubes and evaporated using a SpeedVac concentrator (ThermoFisher). The residue was reconstituted with 150 μL of acetonitrile and spin-filtered through 0.22 μm nylon centrifugal filter units (Costar). LC–MS/MS samples were prepared by combining a 90 μL sample with 10 μL of a 10 μM solution of 4,5-diphenylimidazole. Brains were weighed and homogenized directly in 400 μL of cold acetonitrile using a Cole-Parmer LabGen 7B homogenizer in a 4 mL glass vial. Samples were centrifuged in the same vial for 10 min at 4000 × *g*. 300 μL of the supernatant was transferred to clean tubes and evaporated using a SpeedVac concentrator (ThermoFisher). The residue was reconstituted with 150 μL of acetonitrile, sonicated for 30 s, and spun down at 7500 × *g*. The supernatant was spin-filtered through 0.22 μm nylon centrifugal filter units (Costar). After reconstitution, 90 μL of this solution was combined with 10 μL of 4,5-diphenylimidazole (10 μM in acetonitrile) for analysis. In a separate experiment, blood and brains from untreated animals were combined with known amounts of ETK-II-83 or ETK-III-9 and subjected to the same protocol for the determination of a calibration curve. For LC–MS/MS analysis, see Section 3.9. Concentrations were graphed for each time point and given as mean ± SEM. Pharmacokinetic analysis was performed with a biexponential equation (two-compartment model) for absorption and elimination. The elimination constant was used to determine the half-life. AUC was determined by the trapezoid rule.

### 3.12 Open Field Test

Mice were randomly divided into groups of 16. ETK-II-83 and ETK-III-9 were formulated and administered 60 min before assessing the locomotor activity in an open-field acrylic arena (40 cm (l) × 40 cm (w) × 40 cm (h)) equipped with a transparent Plexiglas wall and a detachable fiber base (Stoelting Co.). Mice were allowed to explore the environment freely for 3 min without motivational constraints while behavior was recorded via an overhead digital USB camera (Stoelting Co.). Between trials, the chamber was sanitized with disinfectants to maintain a sterile environment and eliminate olfactory cues. Automated video tracking software (ANY-maze, version 7.48) was utilized to quantify ambulatory movements and fine motor activity from which parameters such as total distance traveled, speed, and movement/freezing time were derived. Statistical analysis was performed using 1-way ANOVA and Tukey’s multiple comparison test.

### 3.13 Rotarod Assay

Mice were trained to successfully complete a 3 min trial on a rotarod (Omnitech Electronics, Inc.) revolving at a speed of 15 rpm. Formulated ETK-II-83 and ETK-III-9 were given via oral gavage. Mice were placed on the rotarod for 3 min, 60 min after administration. If a mouse fell before 3 min had elapsed, it was placed again on the rotating rod. If a mouse fell a second time, the latency to fall from the rotarod was recorded, or if the mouse successfully completed the 3 min trial, the latency was recorded as 120 s. Data analysis was carried out with GraphPad Prism using a 1-way ANOVA and Tukey’s multiple comparison test (*n* = 16).

### 3.14 Elevated Plus maze^23^

Mice were randomly divided into groups of 12. Sixty minutes after administration, the mice were monitored for 5 min in an elevated plus maze with their movements being monitored by a video tracking system (ANY-maze video-tracking system v7.48; Stoelting Co., IL). The primary measure for assessing anxiolytic-like activity was the percentage of time allocated to the open arms of the maze, with increases in this parameter serving as an indicator of anxiolytic efficacy. Data analysis was carried out with GraphPad Prism using a 1-way ANOVA and Tukey’s multiple comparison test.

### 3.15 Marble Burying Test

Female Swiss Webster mice were randomly divided into groups of ten animals. The marble burying test was conducted with 20 standard glass toy marbles (assorted styles and colors, 15 mm in diameter, 5.2 g in weight) placed gently on a fresh, unscented mouse bedding material filled to a depth of 5 cm and leveled in standard polycarbonate cages (26 cm × 48 cm × 20 cm) with fitted filter-top covers arranged in 5 rows of 4 marbles. One hour after compound administration, each individual mouse was placed into a corner of the cage containing marbles without food and water and allowed to remain in the cage undisturbed for 30 min. After test completion, the number of marbles buried was scored if two-thirds of their surface area was covered by bedding. Data analysis was carried out with GraphPad Prism using a 1-way ANOVA and Tukey’s multiple comparison test.

### 3.16 Statistical Analyses

Statistical analyses were conducted using GraphPad Prism 10 (GraphPad Software, San Diego, CA, USA). For pharmacokinetic analysis, nonlinear regression using a two-compartment model (biexponential equation for absorption and elimination) was employed. For dose–response analysis with nonlinear regression, IC50 ±95% confidence intervals were utilized; for multiple comparisons, one-way analysis of variance (ANOVA) was employed with Tukey’s multiple comparison test. Data are either expressed as either the mean ± SD or mean ± SEM. A value of *P* < 0.05 was considered statistically significant. All P values are indicated in the corresponding figures.

## Supplementary Material



## Notes

NMR data and purity analysis of compounds, receptor binding curves, microsomal stability assay protocol and data, and free drug brain binding protocol (PDF)
