# Supplementary material for: Development of Novel Pegylated Imidazobenzodiazepines Targeting α5 GABAA Receptors for Anxiety: A Comparative Prodrug and Metabolite Study
Source: ACS Chem Neurosci. 2026 Aug 17;17(17):3181–91. doi: 10.1021/acschemneuro.6c00222 (PMC13542478; doi:10.1021/acschemneuro.6c00222)
Supplement: Supplementary file 1 [file cn6c00222_si_001.pdf]

Supporting Information

Development of novel pegylated imidazobenzodiazepines targeting α5 $GABA_A$ receptors for anxiety; a comparative prodrug and metabolite study

Mubaraq A. Toriola,[1] Ethan T. Kowalczyk,[1] Kayode M. Medubi,[1] Michelle J. Meyer,[1] Maya R. T. Fernando,[1] Petra Scholze,[2] Nethyanji Premananda,[1] Shaun G. Harrington,[1] Braden R. Barkley,[1] Daniel A. Webb,[1] and Leggy A. Arnold[1*]

[1]Department of Chemistry and Biochemistry and the Milwaukee Institute for Drug Discovery, University of Wisconsin-Milwaukee, 2000 E. Kenwood Blvd, Milwaukee, Wisconsin 53211, United States

[2]Department of Pathobiology of the Nervous System, Center for Brain Research, Medical University of Vienna, Spitalgasse 4, 1090 Vienna, Austria

Contents

Figure S1: ETK-II-83 ^{1}H NMR spectrum 2

Figure S2: ETK-II-83 ^{12}C NMR spectrum 2

Figure S3: ETK-II-83 ^{19}F NMR spectrum 3

Figure S4: ETK-II-83 Purity analysis 3

Figure S5: ETK-III-9 ^{1}H NMR spectrum 3

Figure S6: ETK-III-9 ^{13}C NMR spectrum 4

Figure S7: ETK-III-9 ^{19}F NMR spectrum 4

Figure S8: ETK-III-9 Purity analysis 4

Figure S9: ETK-II-83 and ETK-III-9 cell viability graph 5

Figure S10: ETK-II-83 and ETK-III-9 $GABA_AR$ binding in the rat brain 5

Figure S11: ETK-II-83 and ETK-III-9 KOR binding 5

Figure S12: ETK-II-83 and ETK-III-9 binding to GABA(A)R subtypes 6

Microsomal stability assay protocol and data (Phase I): 6

Table S1: Data for Microsomal stability assay (phase 1) 7

Metabolic Stability Assay Protocol and data (Phase II: Glucuronidation). 7

Table S2: Data for Microsomal stability assay (phase 2) 8

Figure S13: Time dependent metabolism of ETK-II-83 and formation of ETK-III-9 9

Figure S14: Metabolites of ETK-II-80 10
Figure S15: Metabolites of ETK-II-81 11
Figure S16: Metabolites of ETK-II-83 12
Figure S17: Metabolites of ETK-II-84 13
Table S3: Data for pharmacokinetic data (Areas and concentrations) 14
Free drug brain binding study 14
Table S4: Data for Free drug brain binding 15
References 15

Figure S1: ETK-II-83 ^{1}H NMR spectrum

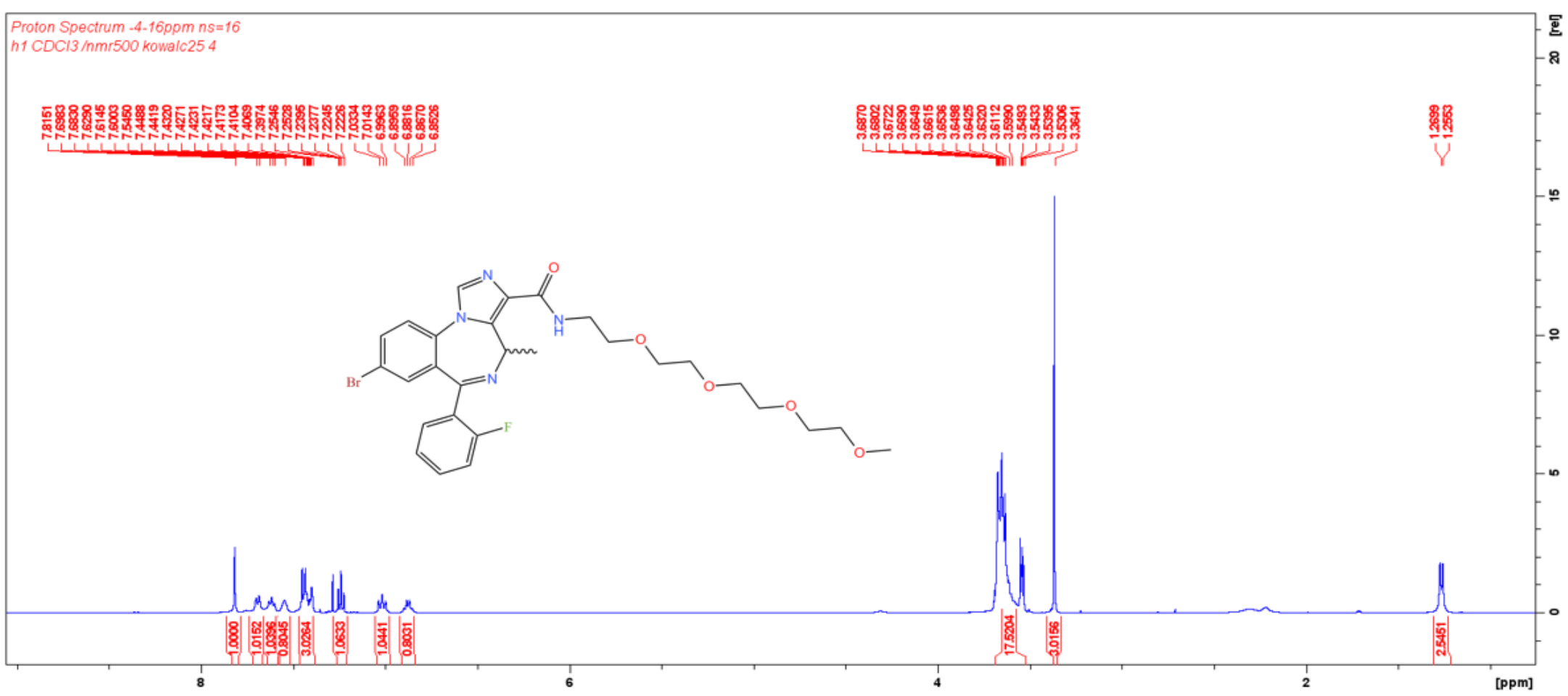

Figure S2: ETK-II-83 ^{12}C NMR spectrum

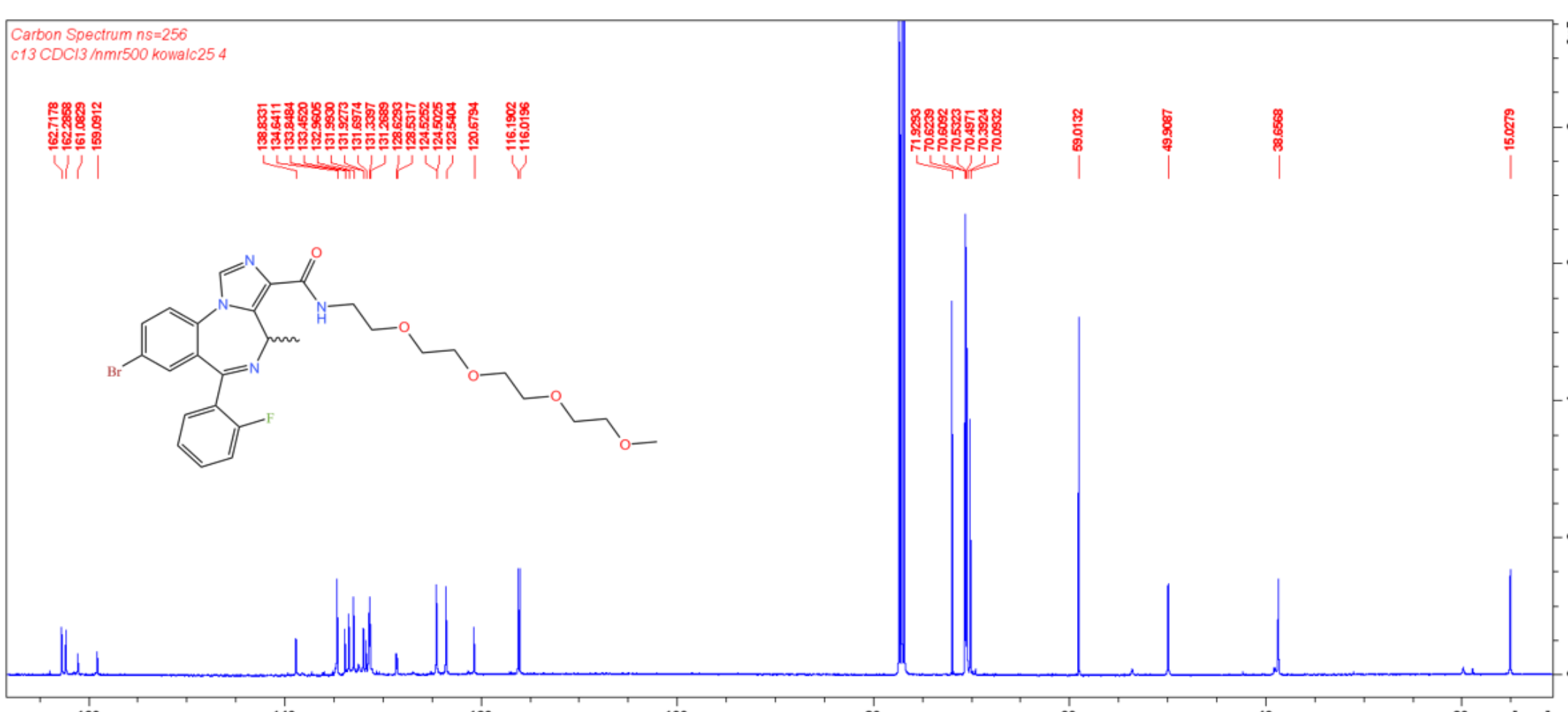

Figure S3: ETK-II-83 ^{19}F NMR spectrum

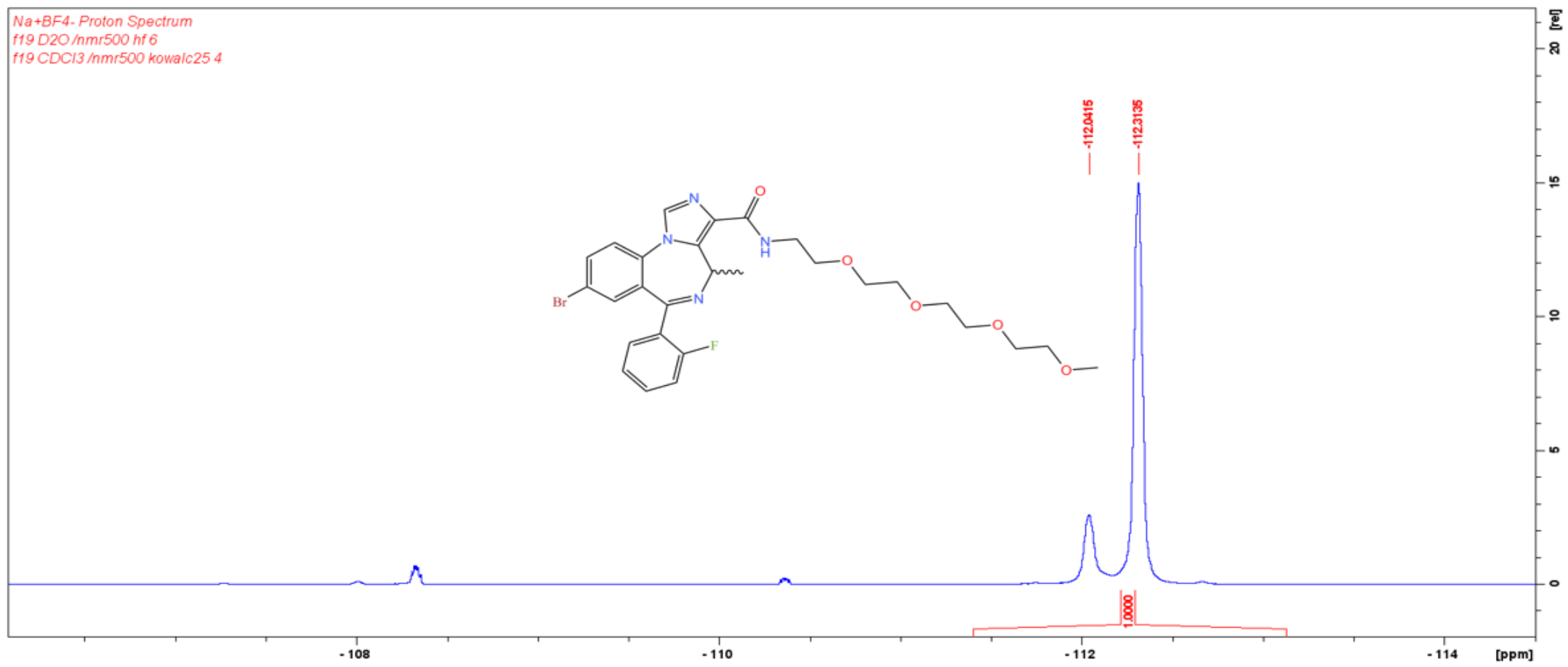

Figure S4: ETK-II-83 Purity analysis

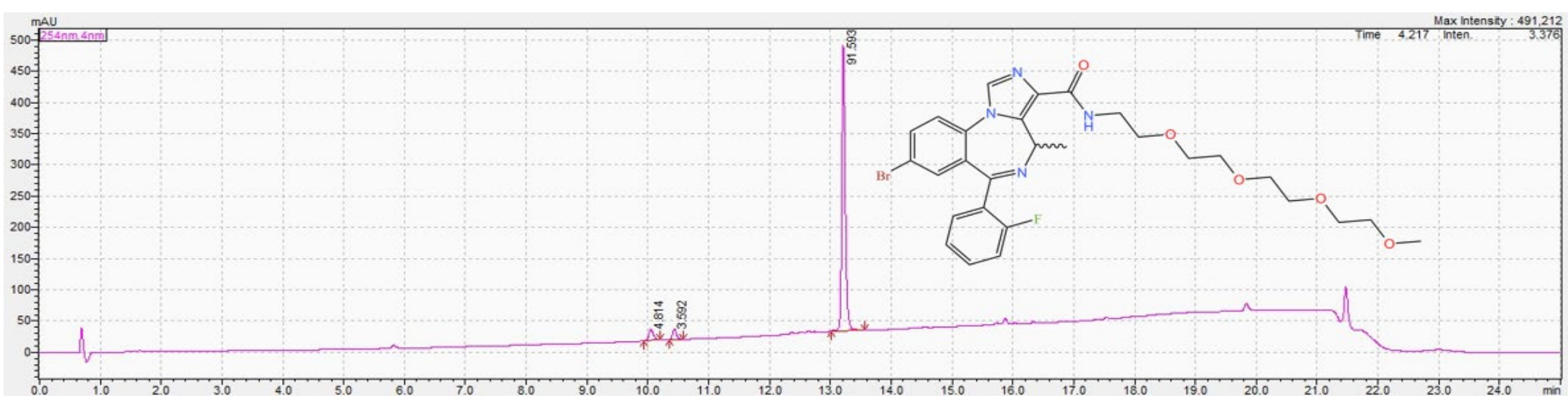

Figure S5: ETK-III-9 ^{1}H NMR spectrum

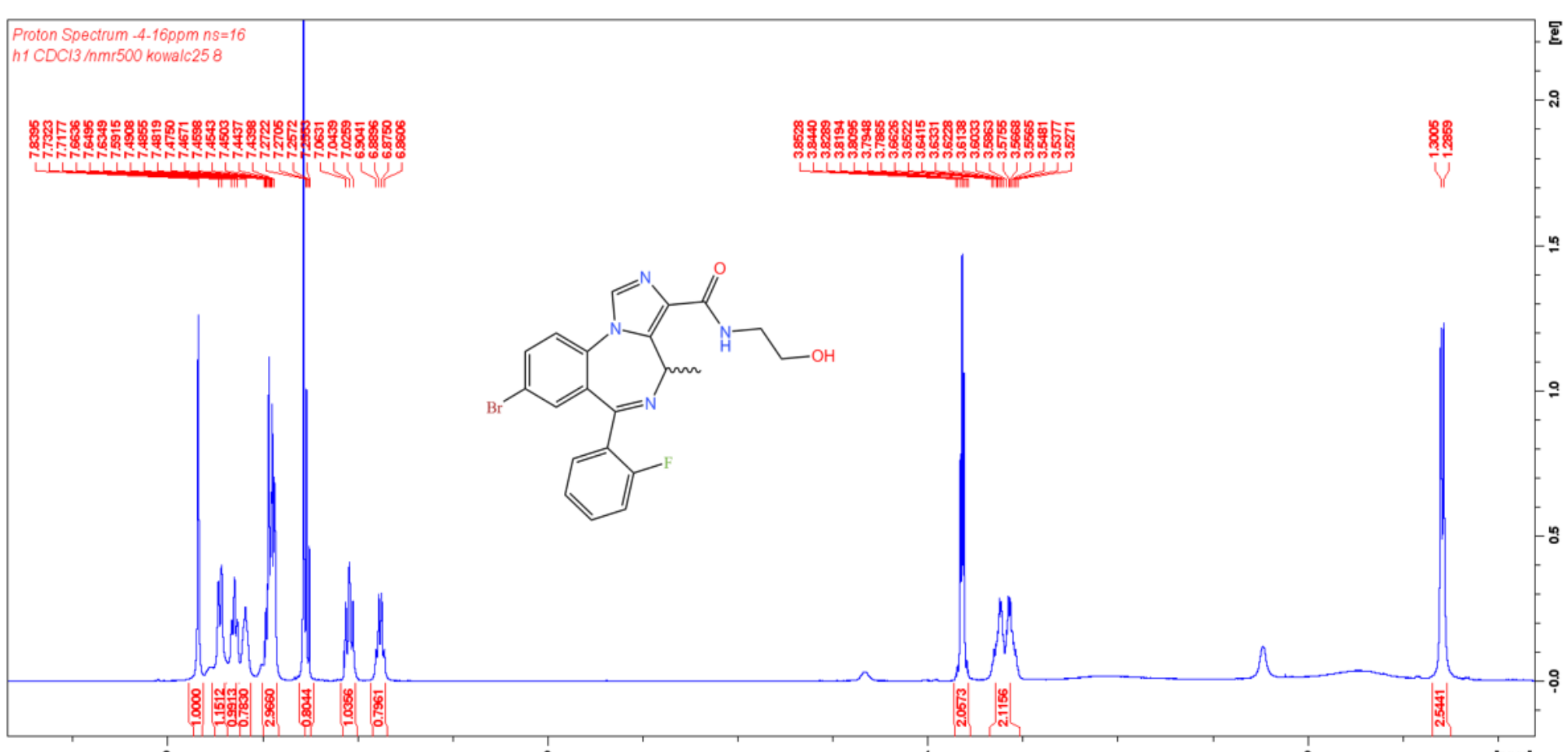

Figure S6: ETK-III-9 ^{13}C NMR spectrum

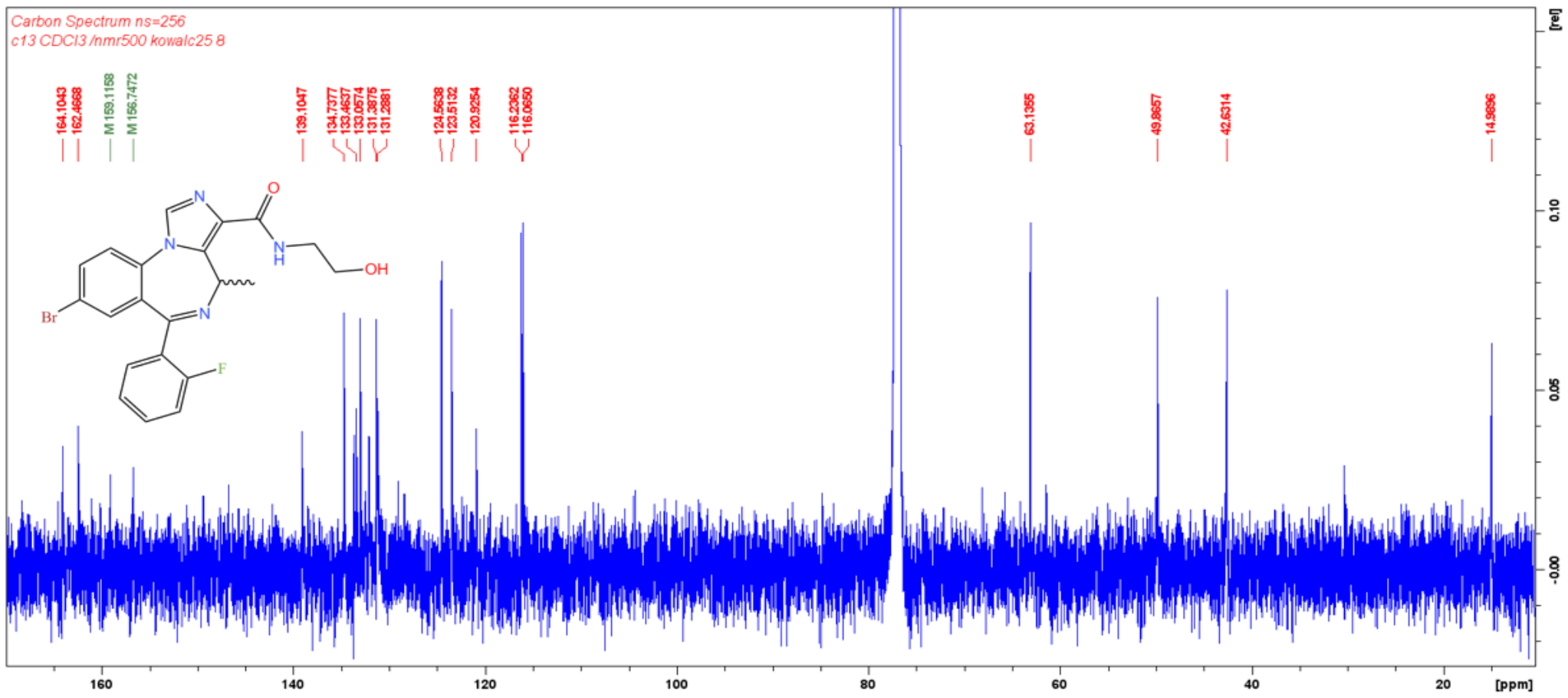

Figure S7: ETK-III-9 ^{19}F NMR spectrum

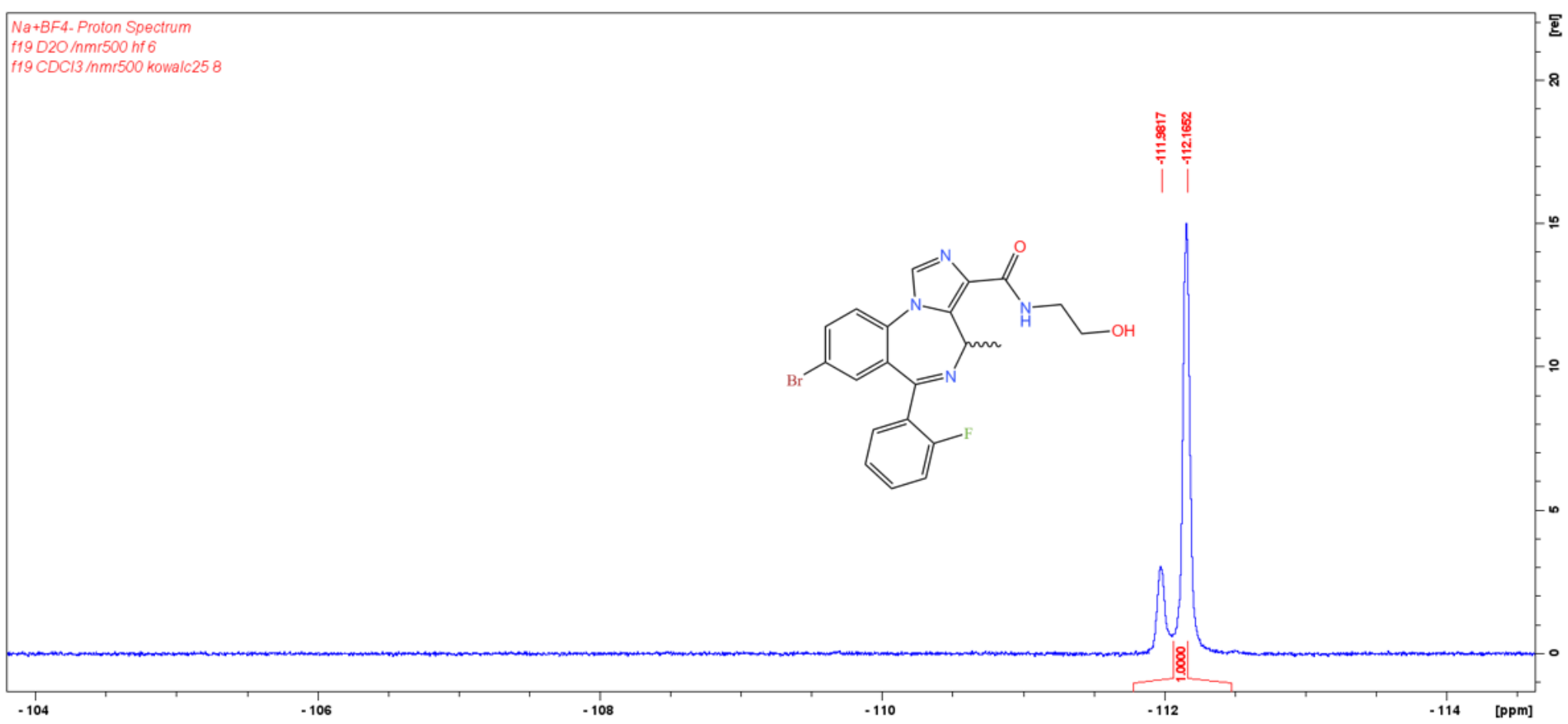

Figure S8: ETK-III-9 Purity analysis

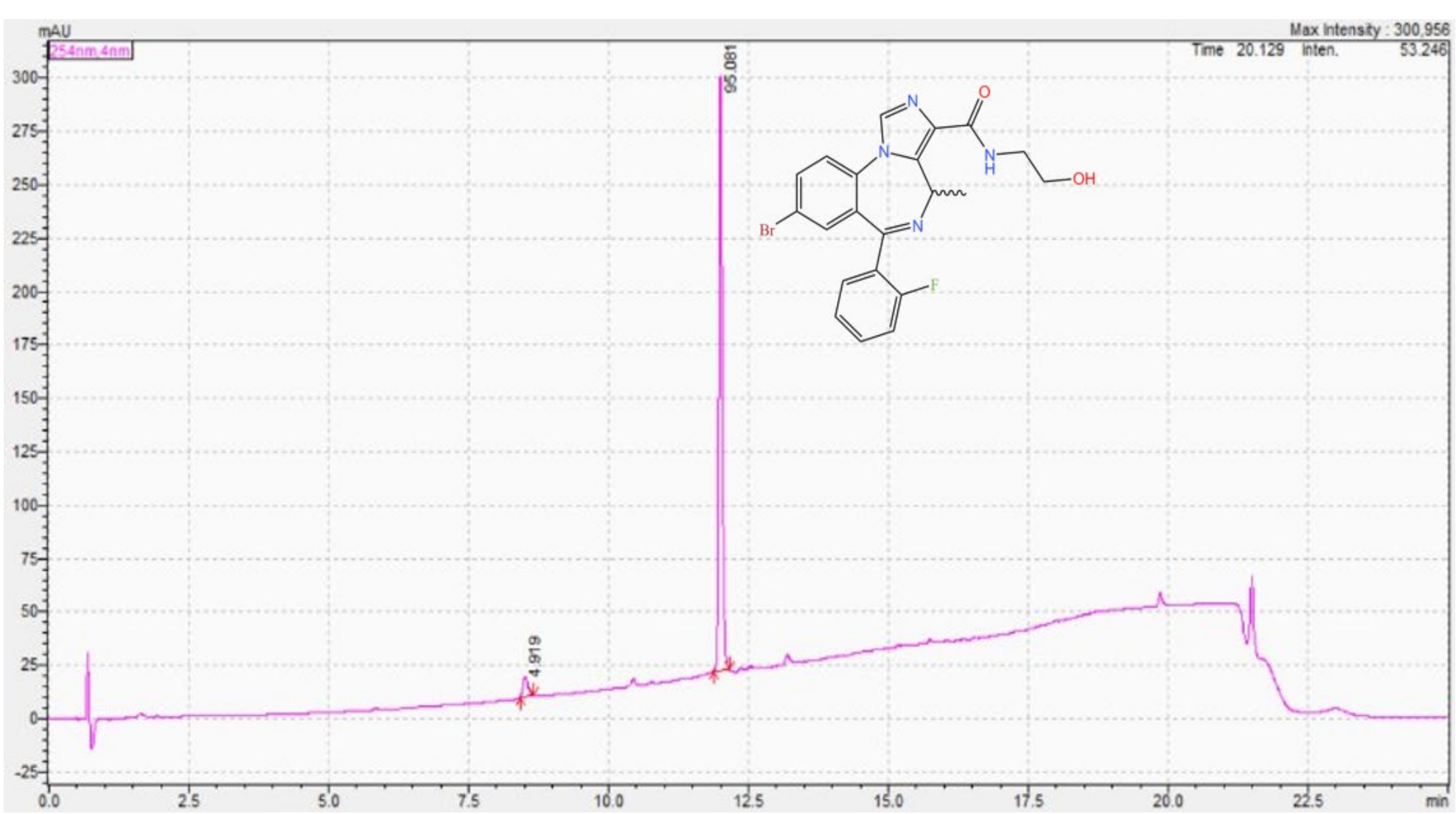

Figure S9: ETK-II-83 and ETK-III-9 cell viability graph

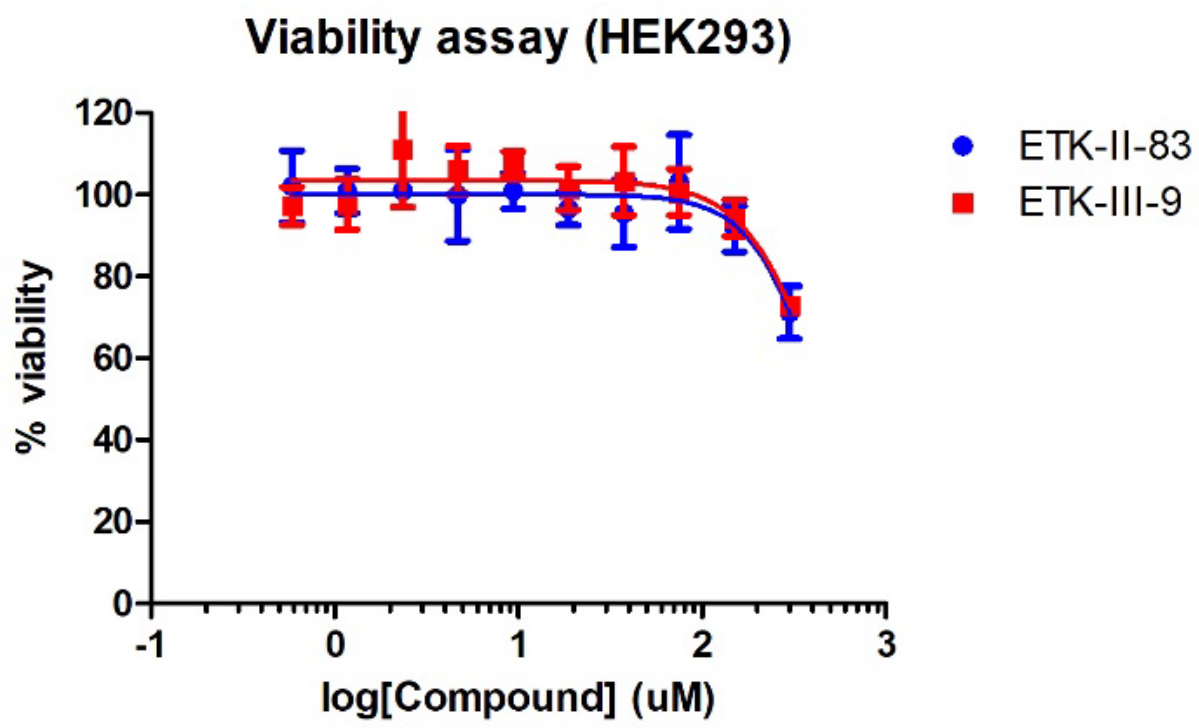

Figure S10: ETK-II-83 and ETK-III-9 $GABA_AR$ binding in the rat brain

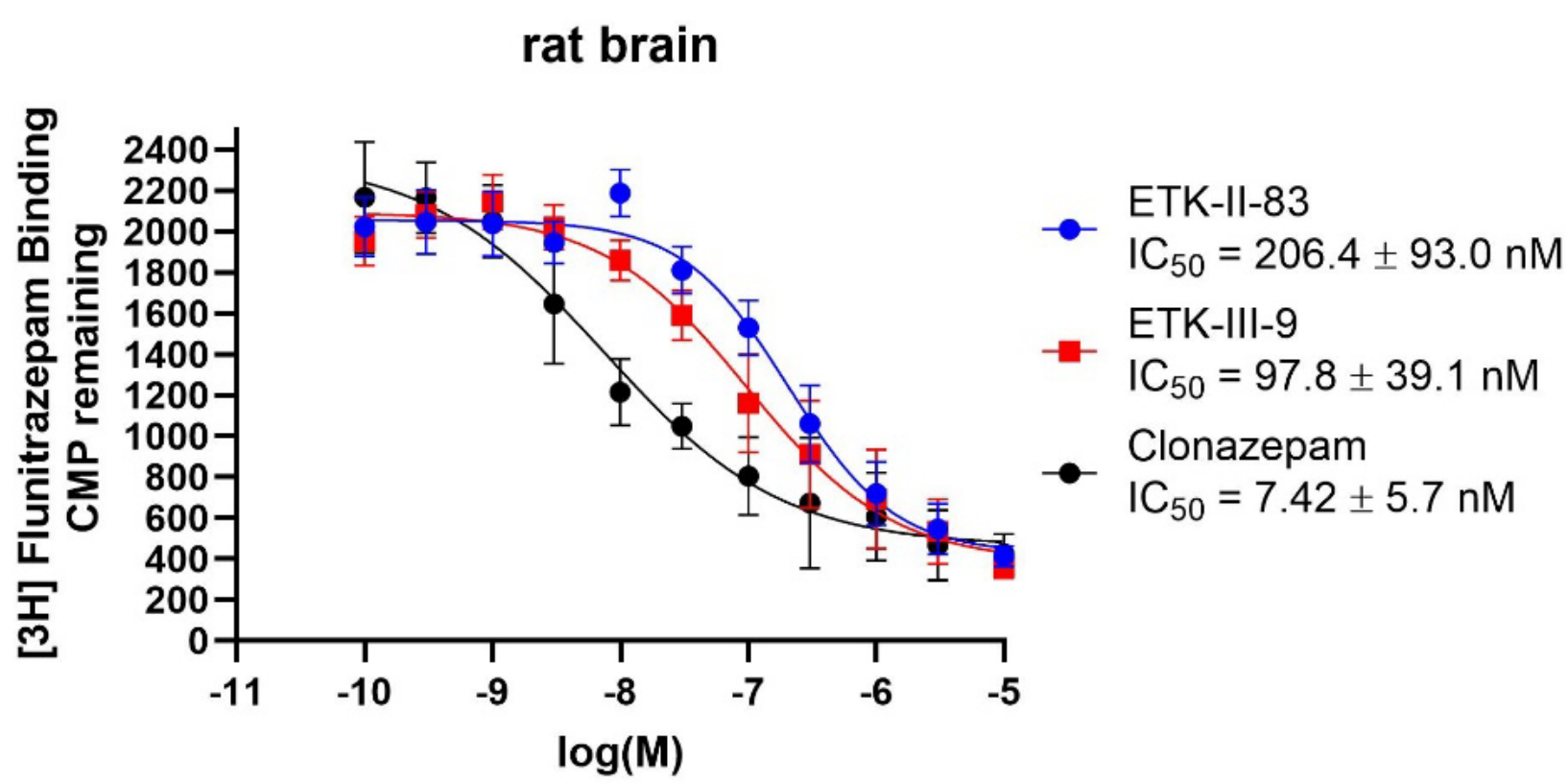

Figure S11: ETK-II-83 and ETK-III-9 KOR binding

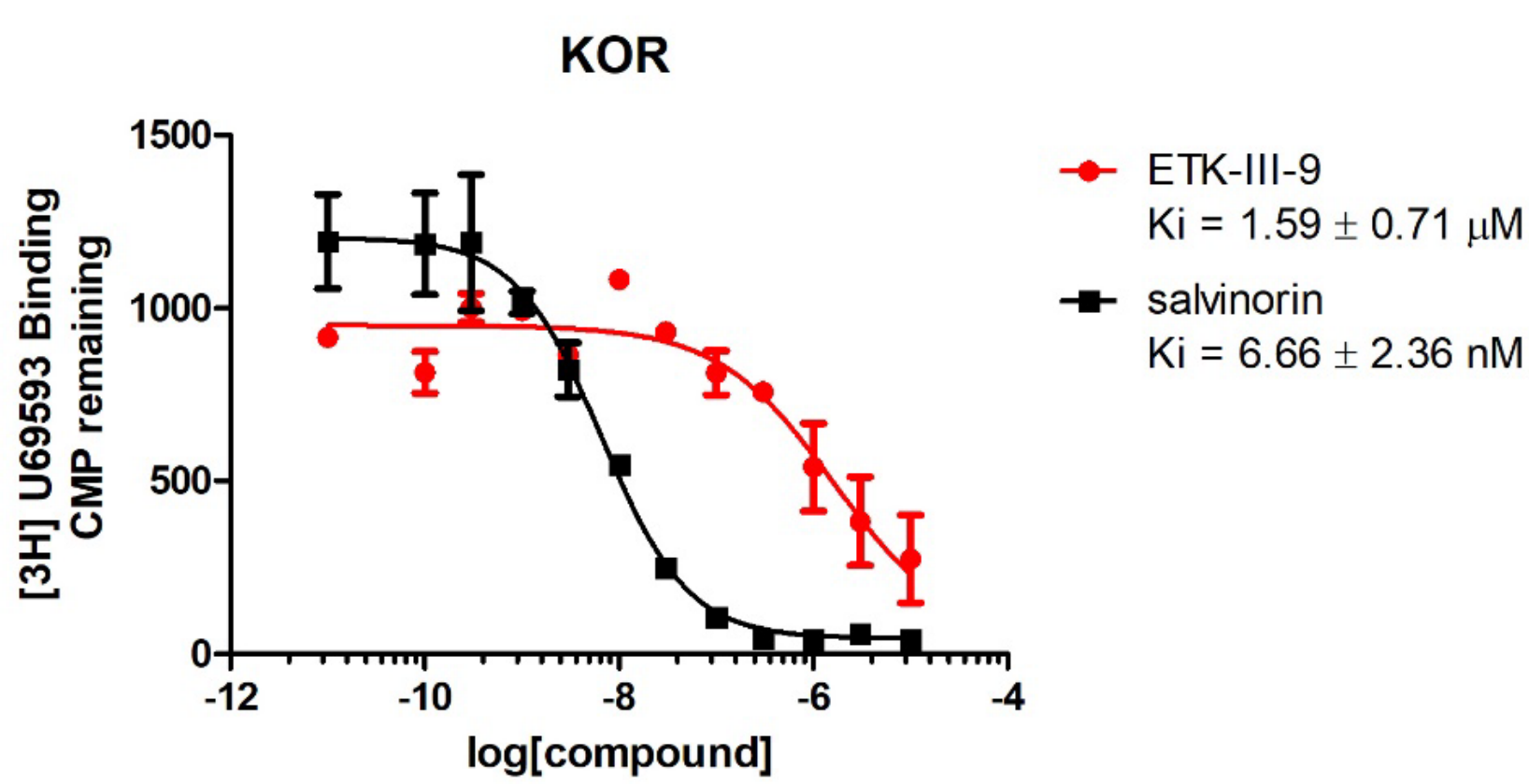

Figure S12: ETK-II-83 and ETK-III-9 binding to GABA(A)R subtypes

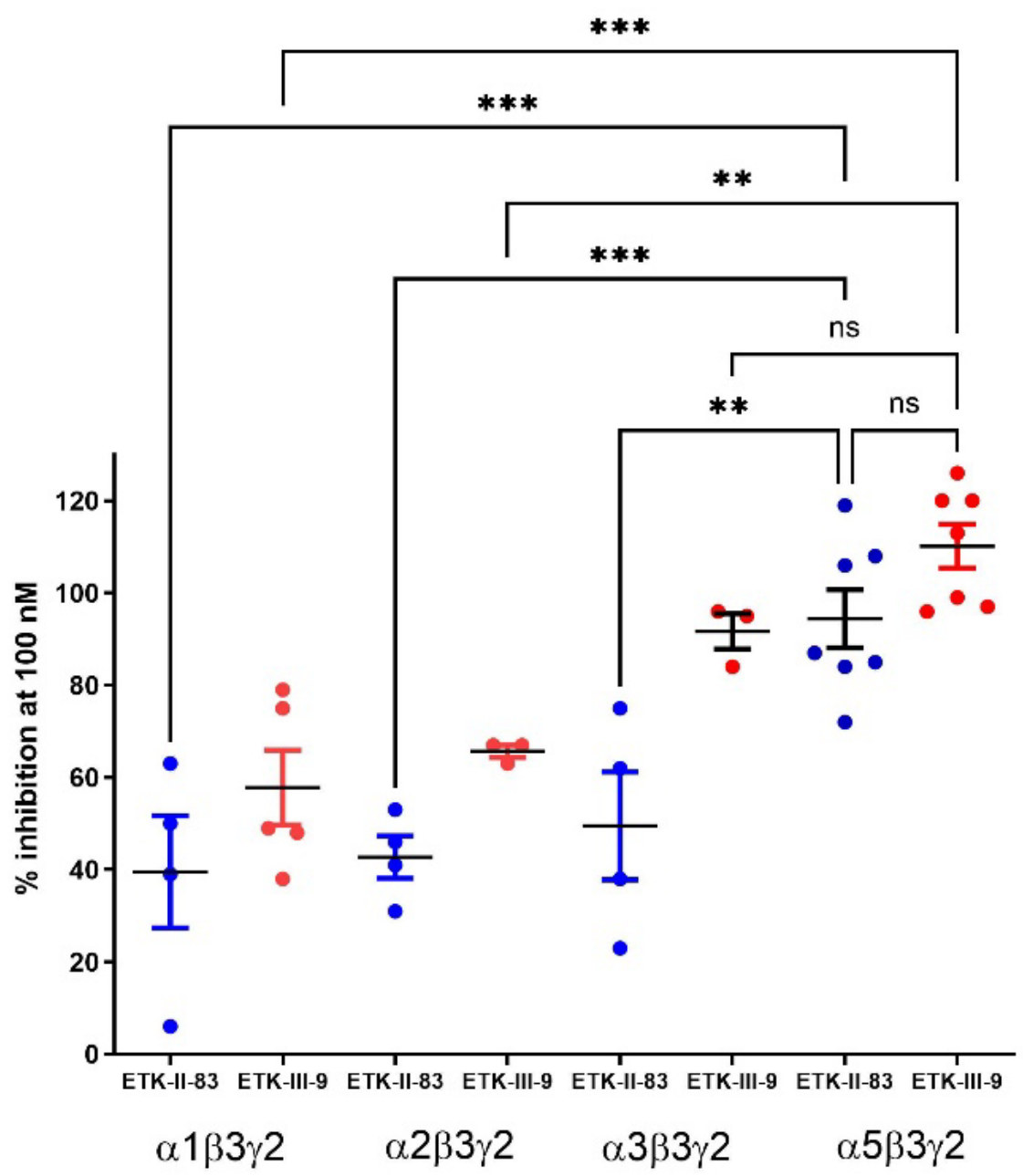

Significances were calculated with 1-way ANOVA using Tukey's multiple comparison test: n.s. $p > 0.5$, *$p < 0.05$, **$p < 0.01$, and ***$p < 0.001$.

Microsomal stability assay protocol and data (Phase I):

4 µL of 1 mM test compound in DMSO was added to a mixture of 282 µL water, 80 µL phosphate buffer (0.5 M, pH 7.4), 20 µL NADPH Regenerating System Solution A (BD Bioscience, San Jose, CA), and 4 µL NADPH Regenerating System Solution B (BD Bioscience). The assay concentration of test compound was 10 µM. The solution was pre-incubated at 37°C for 5 min using a digital heating shaking dry bath (Fischer Scientific, Pittsburgh, PA), followed by removal of 50 µL at the 0 min time point, which was added to 100 µl methanol that contained 10 µM 4,5-diphenylimidazole as internal standard (IS). The reaction was initiated by adding 8.8 µL of mouse liver microsomes (from Xenotech, Kansas City, KS), resulting in a protein concentration of 0.5 mg/mL. 50 µL aliquots were taken at 120 minutes. Like the 0 min time point, each aliquot was transferred to 100 µL of cold methanol containing 10 µM IS. This was followed by sonication for 10 sec and centrifugation at 11,000 x g for 5 min. The supernatant was transferred into a spin-X HPLC filter tube (Corning Inc., Corning, NY) and centrifuged at 11,000 x g for 30 sec. The 20 µL filtrate was diluted with 80 µL of solvent; this step was modified to reduce the volume used and subsequently analyzed by LC-MS/MS with Shimadzu LCMS 8040 mass spectrometer (Shimadzu, Kyoto, Japan). The ratio of the peak areas of the internal standard and the test compounds was calculated for every time point. The remaining percentage was determined using the area ratio of the 120 min time point and the start time (T0). All determinations were carried out on two different days in triplicate. (n = 6) The activity of microsomes was tested with reference compound HZ166 for phase 1.[1]

Analysis was conducted with a Shimadzu Nexera X2 LC30AD (Shimadzu, Kyoto, Japan) using a 5 µL injection volume. Separation was achieved by reverse-phase chromatography using an Eclipse plus II RRHD Extend-C18. A 2.1 mm x 50 mm column with 1.8 µm particle size was used with a 0.4 mL/min flow rate. The mobile phase was methanol and water (both containing 0.1% formic acid). Program: 35% B (0 min) → 99% B (4 min), 99% B (4 min), → 99% B (2 min), hold at 99% B (2 min), return to 35% B (0.10 min), hold at 35% B (1 min). The column temperature was 25 °C. Analysis was performed with positive ionization using MRM (multiple reaction monitoring) with a Shimadzu 8040 (Shimadzu, Kyoto, Japan) using electrospray, as well as atmospheric pressure ionization (DUIS). The following transitions were monitored in MRM mode Ion pairs for ETK-II-83 were m/z 603.10 > 439.00, m/z 603.10 > 396.00, m/z 603.10 > 421.95; Ion pairs for ETK-III-9 were m/z 457.00 > 396.00, m/z 457.00 > 368.00, 457.00 > 326.90; Internal standard m/z 221.00 > 194.15, m/z 221.00 > 165.15, m/z 221.00 > 91.05. The collision energy was adjusted for each ion transition. The heating block temperature was 400°C. The drying gas flow was 15 L/min and the desolvation line temperature was 250°C. The nebulizing gas flow was 1.5 L/min and a voltage of 4.5 kV was used for the needle and interface. Data acquisition and processing were performed using LabSolutions software.

Table S1: Data for Microsomal stability assay (phase 1)

Time 0	Area of Compound:	7,607,562	5.307499	0.837182	
	Area of DPI:	1,433,361			83.72
Time 120	Area of Compound:	7,077,815	4.443343		
	Area of DPI:	1,592,903			
Time 0	Area of Compound:	9,254,631	5.624708	0.886554	
	Area of DPI:	1,645,353			88.66
Time 120	Area of Compound:	8,168,215	4.986609		
	Area of DPI:	1,638,030			
Time 0	Area of Compound:	9,030,184	4.506687	0.873724	
	Area of DPI:	2,003,730			87.37
Time 120	Area of Compound:	6,445,984	3.937602		
	Area of DPI:	1,637,033			
Time 0	Area of Compound:	9,004,495	5.384417	0.930403	
	Area of DPI:	1,672,325			93.04
Time 120	Area of Compound:	8,253,471	5.009676		
	Area of DPI:	1,647,506			
	average	88.20			
	SD	3.847372			

Time 0	Area of Compound:	15,127,167	2.580604	0.034802	
	Area of DPI:	5,861,871			3.48
Time 120	Area of Compound:	487,371	0.089809		
	Area of DPI:	5,426,727			
Time 0	Area of Compound:	14,129,908	2.373768	0.0146	
	Area of DPI:	5,952,522			1.46
Time 120	Area of Compound:	190,309	0.034658		
	Area of DPI:	5,491,112			
Time 0	Area of Compound:	13,903,887	2.598586	0.028574	
	Area of DPI:	5,350,558			2.86
Time 120	Area of Compound:	435,266	0.074251		
	Area of DPI:	5,862,100			
	average	2.60			
	SD	1.034521			

Metabolic Stability Assay Protocol and data (Phase II: Glucuronidation).

To 222 µL water, 80 µL phosphate buffer (0.5 M, pH 7.4), 40 µL of a 50 mM of UDP-glucuronic acid in water (5 mM final concentration), 40 µL of a 50 mM solution of inhibitor sacharic-1,4-lactone in water (5 mM final concentration), 4 µL of a 100 mM $MgCl_2$ solution in water (1 mM final concentration), 4 µL of a 1 mM DMSO solution of test compound (10 µM assay concentration), and 1.8 µL of a 5 mg/ml alamethicin in DMSO (0.0225 mg/ml final concentration) were added. The assay was preincubated at 37 °C for 5 min using a heating shaking dry bath (Fischer Scientific), followed by a 50 µL aliquot being removed and quenched with 100 µL of cold methanol that contained 5 µM 4,5-diphenylimidazole as internal standard (IS). 8.8 µl of mouse liver S9 fraction (Xenotech) was added

to initiate the reaction. The protein assay concentration was 0.5 mg/ml. A final aliquot was taken after 120 min and quenched with 100 µL of cold methanol containing 5 µM IS. The samples were sonicated for 10 s, centrifuged at 11,000 x g for 5 min, and filtered using a 0.22 µm nylon spin-X HPLC filter tube (Corning Inc.), and centrifuged at 11,000g for 30 s. For the analysis by LC-MS/MS (Shimadzu), the samples were diluted 10-fold. The parameters for the instrument analysis can be found in the description of the pharmacokinetics protocol in the manuscript. Peak area ratios between compound area and IS area were used to determine conversion between t = 0 and t = 120 min. The experiments were carried out in two independent assays in triplicate. (n = 6) The activity of microsomes was tested with reference compound MIDD0301 for phase 2.[2]

Table S2: Data for Microsomal stability assay (phase 2)

Time 0	Area of Compound:	2,340,405		6.062738	0.932794		
	Area of DPI:	386,031					
						**93.28**	
Time 120	Area of Compound:	2,238,968		5.655288			
	Area of DPI:	395,907					
Time 0	Area of Compound:	2,417,329		6.494513	0.947307		
	Area of DPI:	372,211					
						**94.73**	
Time 120	Area of Compound:	2,218,414		6.152298			
	Area of DPI:	360,583					
Time 0	Area of Compound:	2,483,133		6.47391	0.966288		
	Area of DPI:	383,560					
						**96.63**	
Time 120	Area of Compound:	2,243,561		6.255659			
	Area of DPI:	358,645					
Time 0	Area of Compound:	2,345,870		6.203019	0.98917		
	Area of DPI:	378,182					
						**98.92**	
Time 120	Area of Compound:	2,255,731		6.13584			
	Area of DPI:	367,632					

Figure S13: Time dependent metabolism of ETK-II-83 and formation of ETK-III-9

t=5						ETK_III_9						
		Compound	DPI	Compound/DPI								
	1	10,818,603	5,855,729	1.869362871		610,274	5,855,729	0.104218	2004.959	181.0583	6014.876	543.175
	2	10,444,520	6,138,890	1.848819286		667,931	6,138,890	0.108803	1982.133	188.4296	5946.398	565.2888
	3	10,095,717	5,814,215	1.775402946		639,179	5,814,215	0.109934	1900.559	190.2473	5701.676	570.742
								0.107652				
t=15						ETK_III_9						
		Compound	DPI	Compound/DPI								
	1	4,261,016	5,787,321	0.704614854		1,359,782	5,787,321	0.234959	710.7943	391.2521	2132.383	1173.756
	2	3,732,683	5,649,292	0.689193114		1,306,745	5,649,292	0.231311	693.659	385.3879	2080.977	1156.164
	3	3,685,706	5,686,437	0.643734995		1,270,536	5,686,437	0.223433	643.15	372.7214	1929.45	1118.164
								0.229901				
t=30						ETK_III_9						
		Compound	DPI	Compound/DPI								
	1	2,010,269	6,047,298	0.33242433		1,951,088	6,047,298	0.322638	297.2493	532.2154	891.7478	1596.646
	2	2,198,775	5,416,019	0.40597623		1,741,743	5,416,019	0.321591	378.9736	530.5322	1136.921	1591.596
	3	2,121,746	5,725,502	0.37057816		1,873,981	5,725,502	0.327304	339.6424	539.7174	1018.927	1619.152
								0.323844				
t=60						ETK_III_9						
		Compound	DPI	Compound/DPI								
	1	654,935	5,733,080	0.114237897		2,315,156	5,733,080	0.403824	54.81989	662.7397	164.4597	1988.219
	2	781,472	5,816,213	0.134360966		2,291,452	5,816,213	0.393977	77.17885	646.9078	231.5366	1940.723
	3	625,871	5,536,305	0.113048504		2,308,949	5,536,305	0.417056	53.49834	684.0128	160.495	2052.038
								0.404952				
t=120						ETK_III_9						
		Compound	DPI	Compound/DPI								
		333,780	6,281,313	0.053138572		2,982,461	6,281,313	0.474815	13.06825	776.8728	39.20476	2330.618
		379,339	5,840,195	0.064953139		2,613,159	5,840,195	0.447444	0.059044	732.8678	0.177131	2198.604
		288,141	5,788,539	0.049777845		2,764,140	5,788,539	0.477519	16.80239	781.221	50.40718	2343.663
								0.466593				

Figure S14: Metabolites of ETK-II-80

ETK-II-80

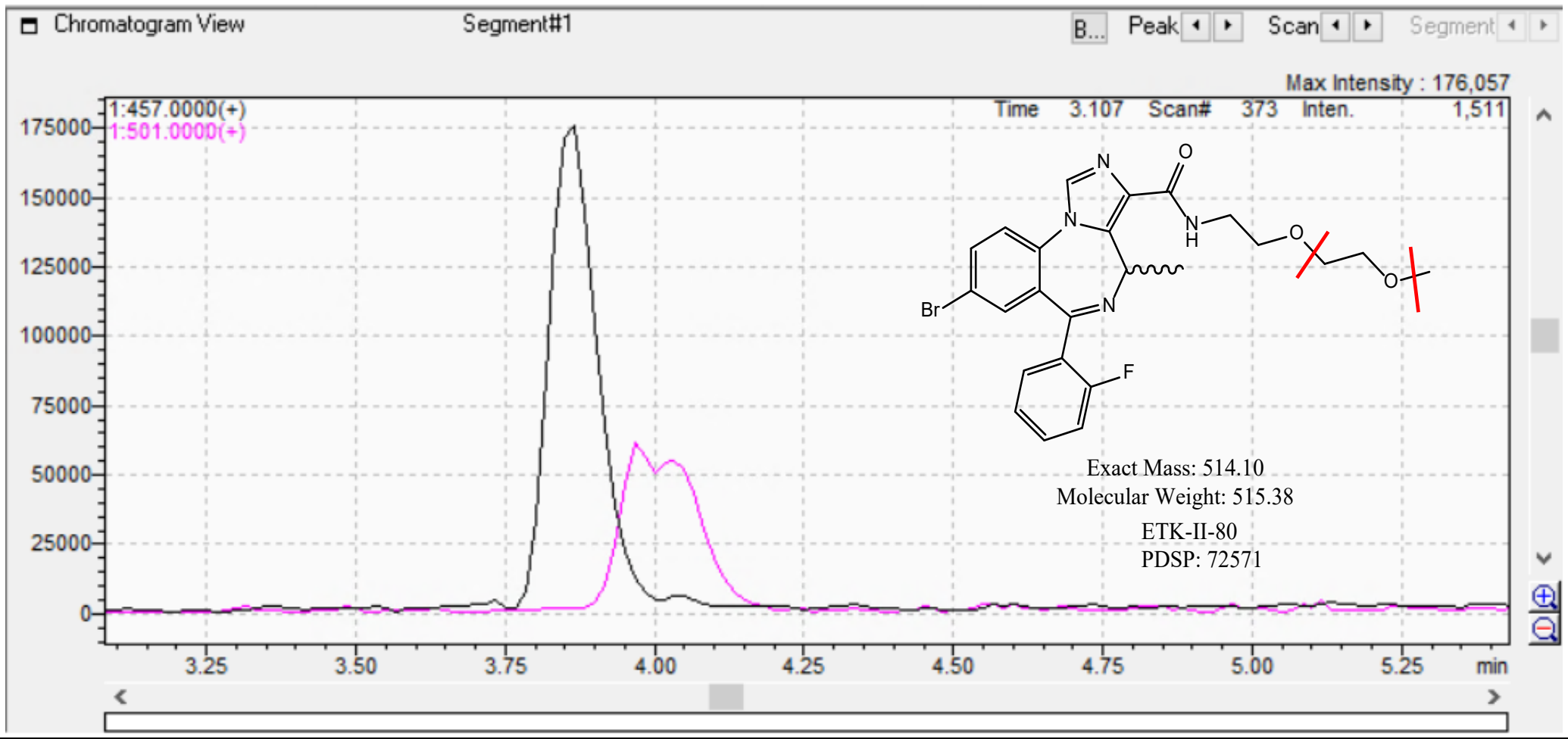

Metabolite m/z	Screenshot	Retention Time (min)	Peak Area	%
457	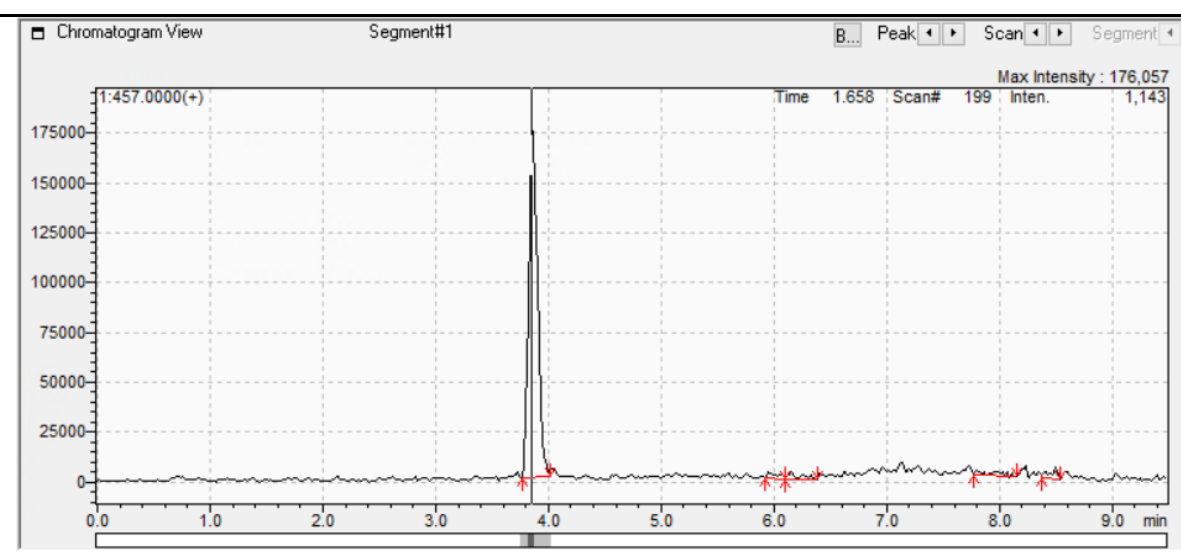	3.861	982844	80.2 %
501	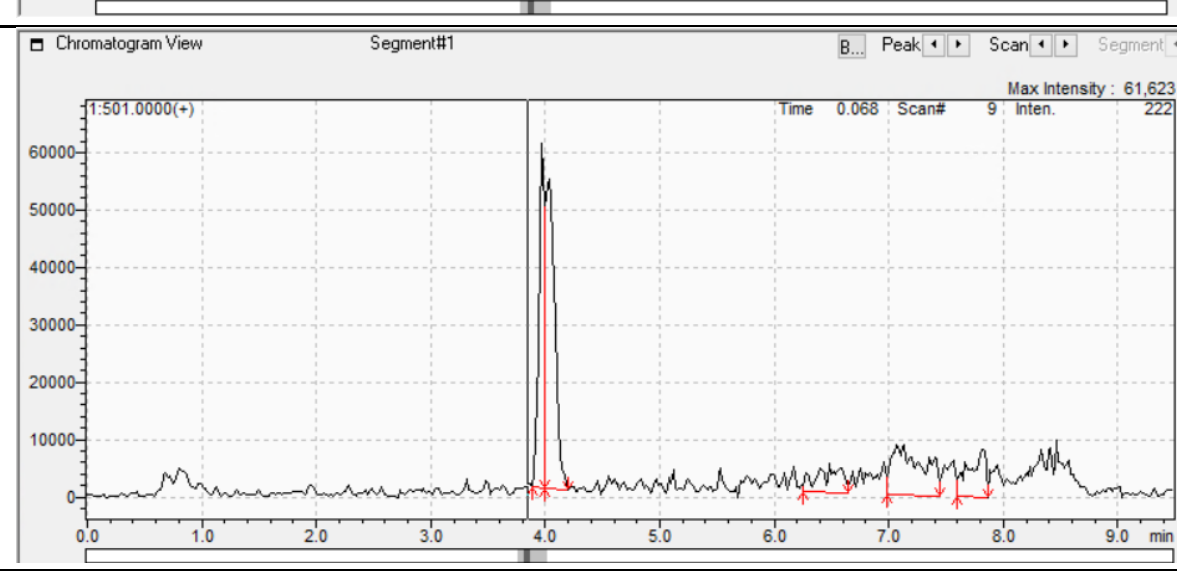	3.971	242243	19.8 %
Total			1225087	

Figure S15: Metabolites of ETK-II-81

ETK-II-81

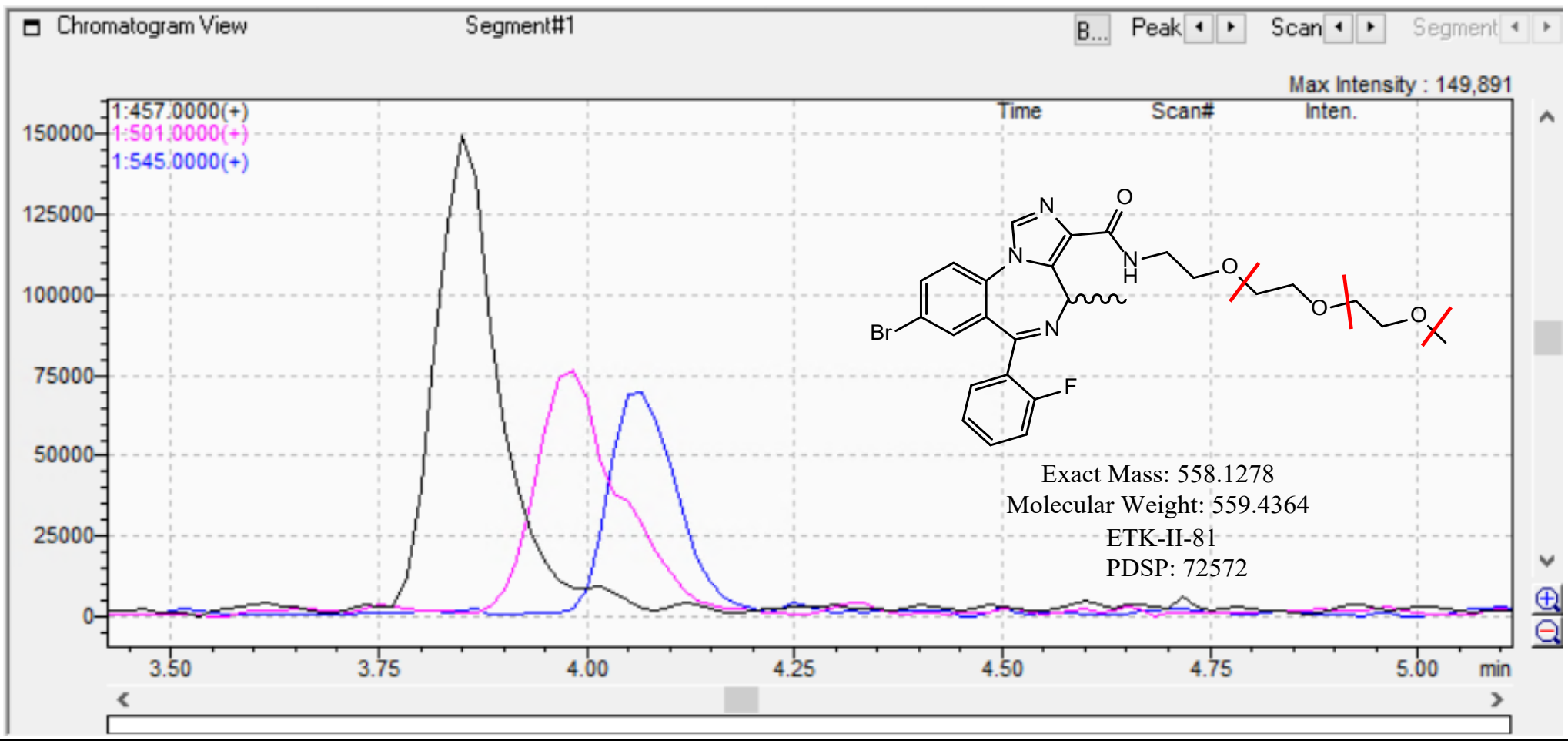

Metabolite m/z	Screenshot	Retention Time (min)	Peak Area	%
457	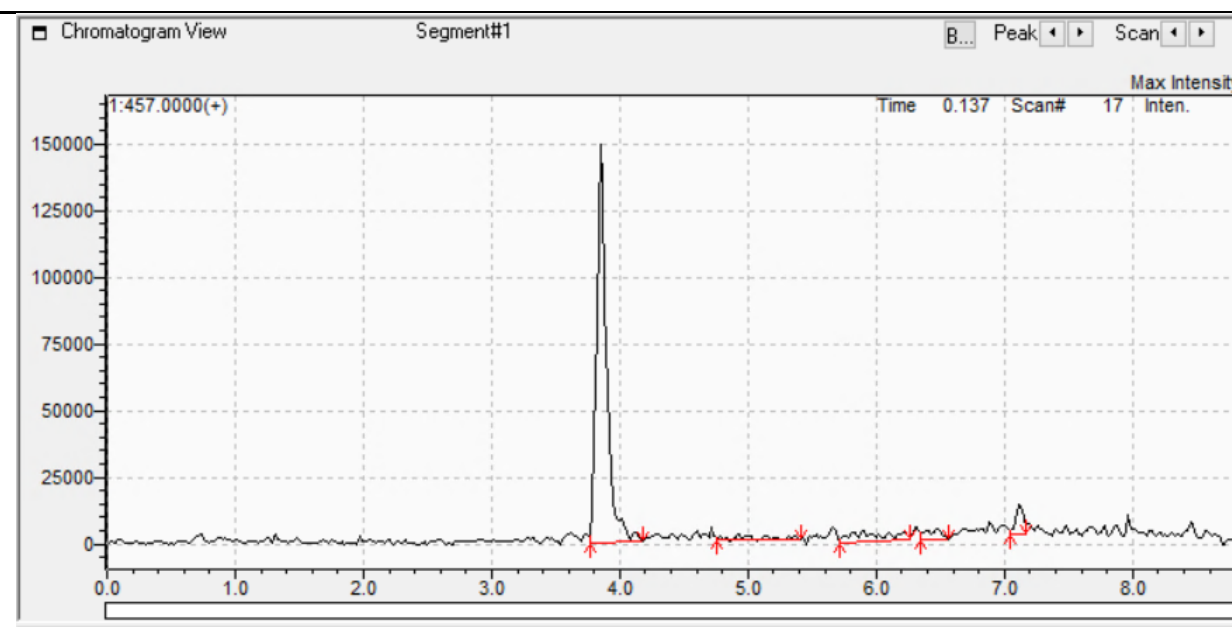	3.853	814719	46.5 %
501	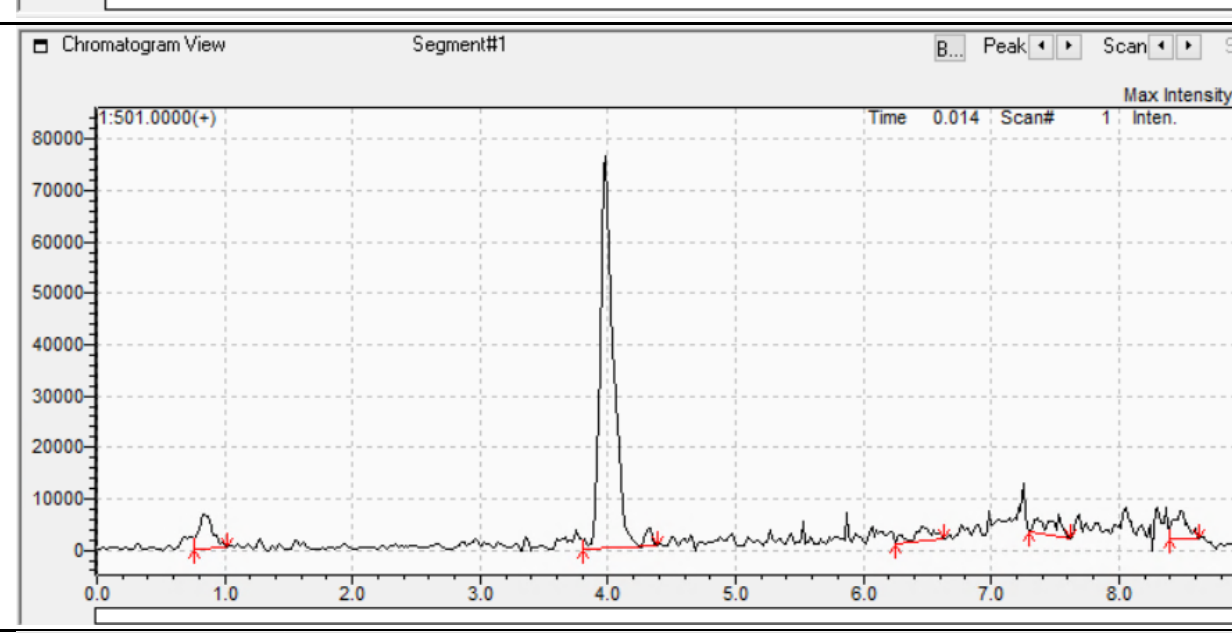	3.979	549671	31.4 %
545	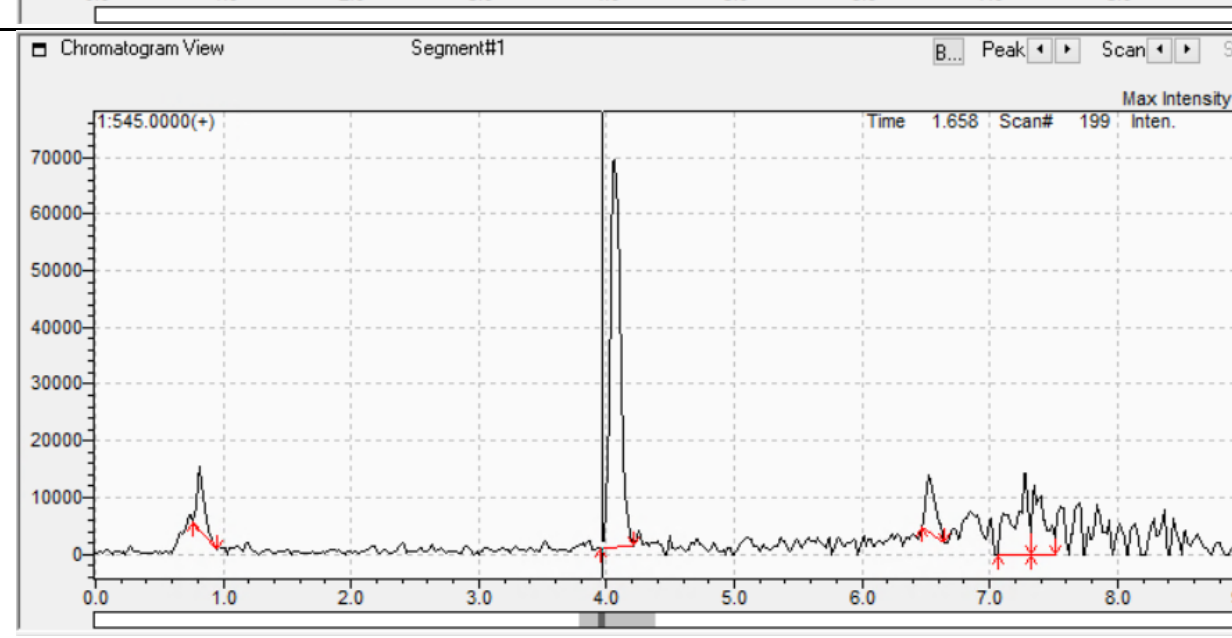	4.059	386808	22.1 %
Total			1751198	

Figure S16: Metabolites of ETK-II-83

ETK-II-83

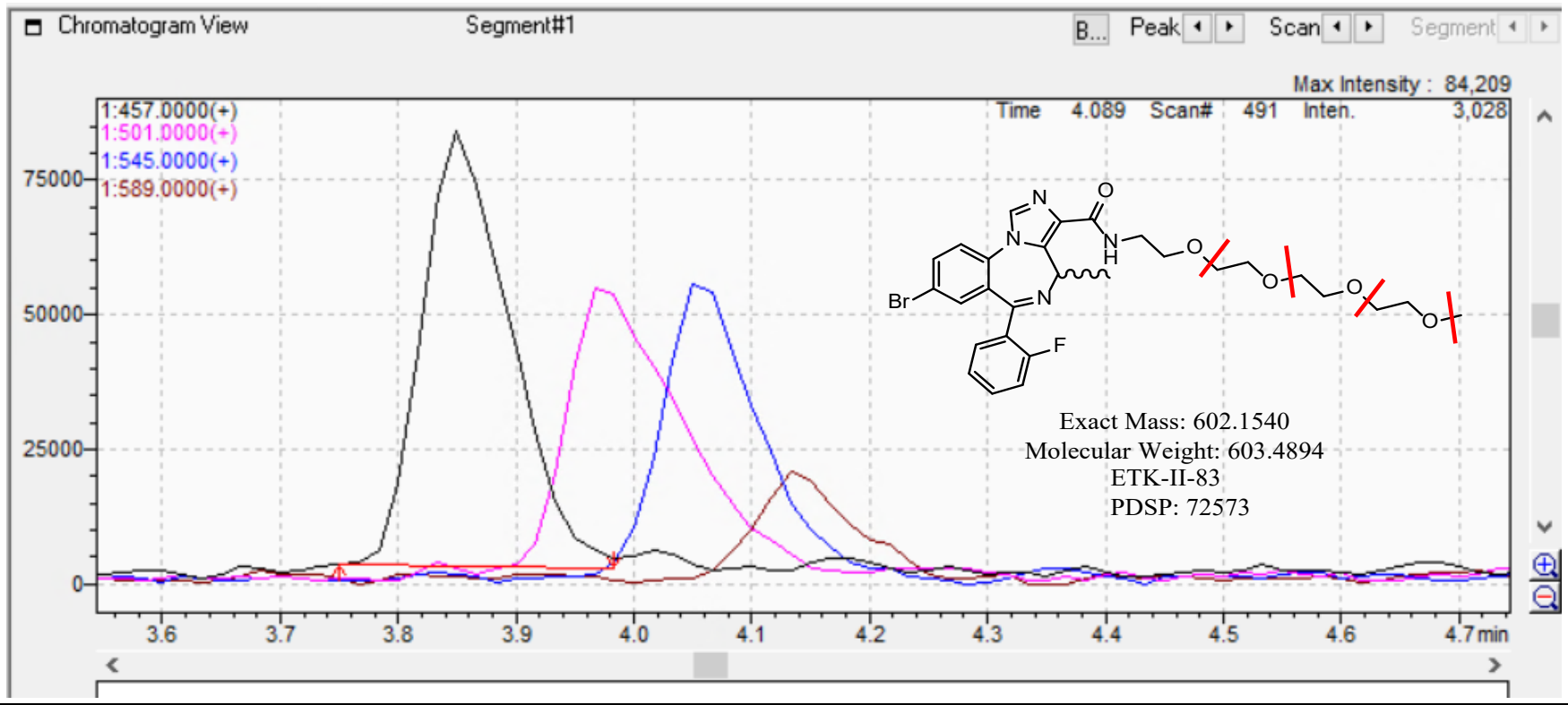

Metabolite m/z	Screenshot	Retention Time (min)	Peak Area	%
457	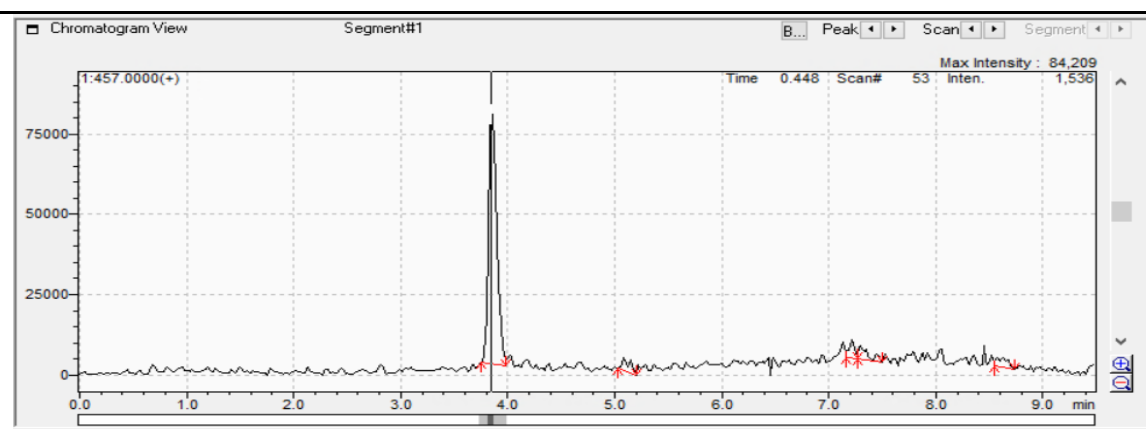	3.851	420528	34.3%
501	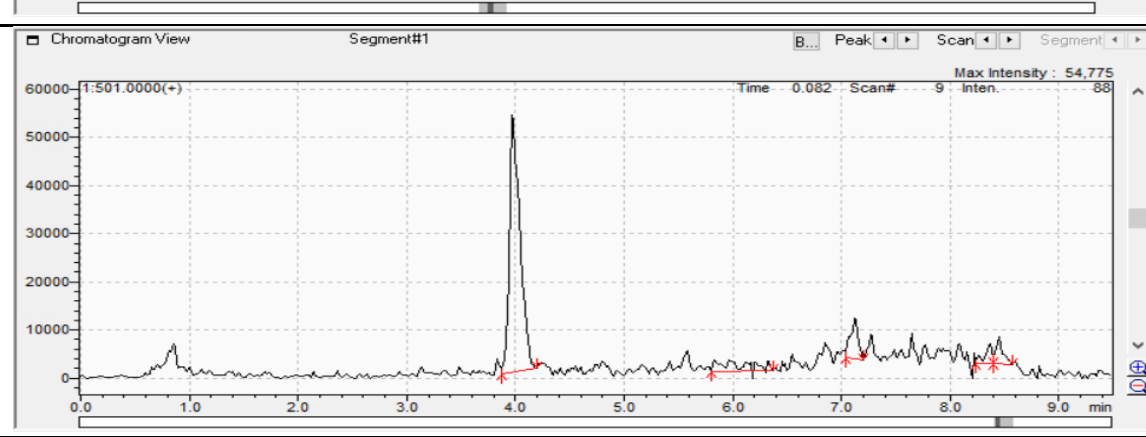	3.974	372009	30.4%
545	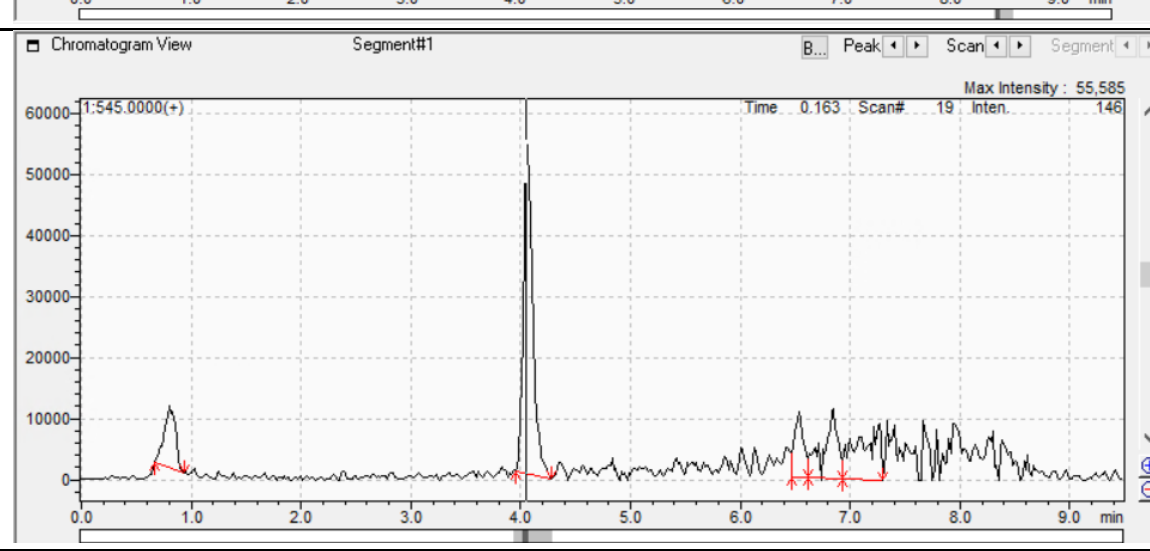	4.057	320681	26.2%
589	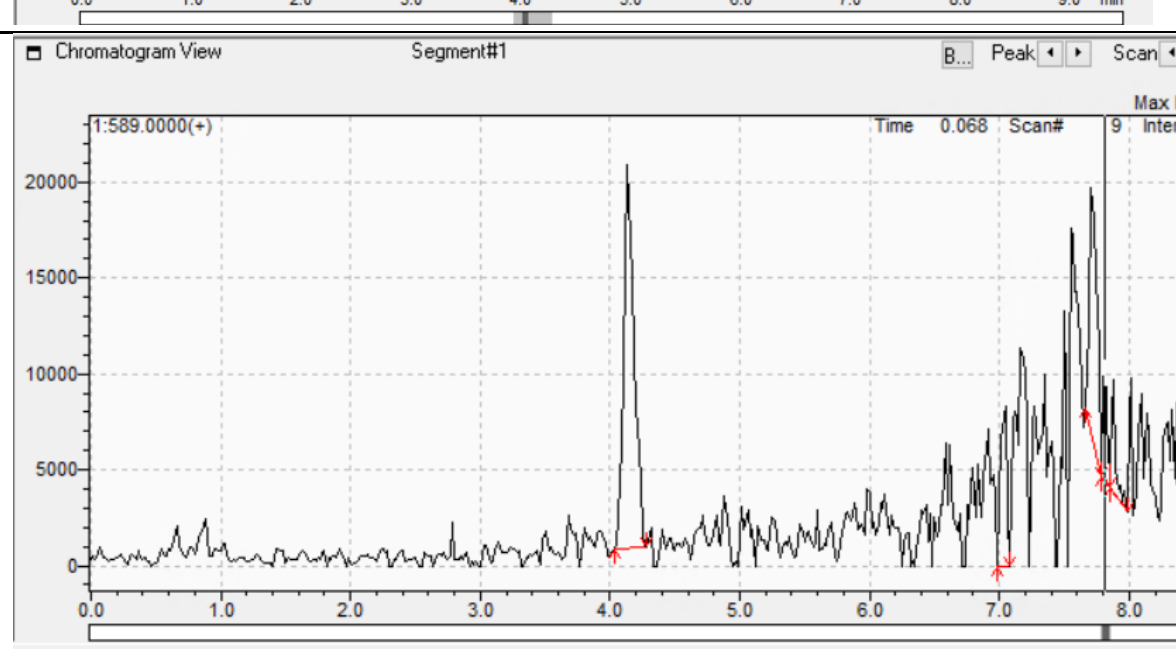	4.137	111708	9.1%
			1224926	

Figure S17: Metabolites of ETK-II-84

ETK-II-84

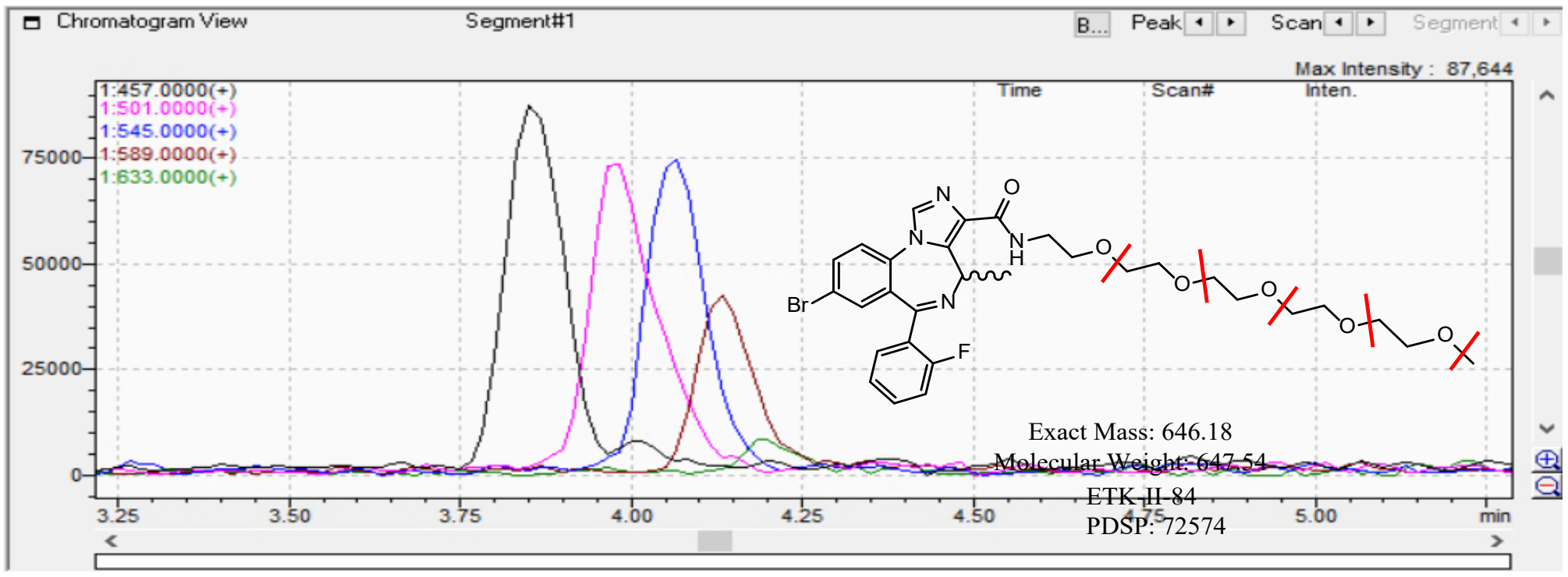

Metabolite m/z	Screenshot	Retention Time (min)	Peak Area	%
457	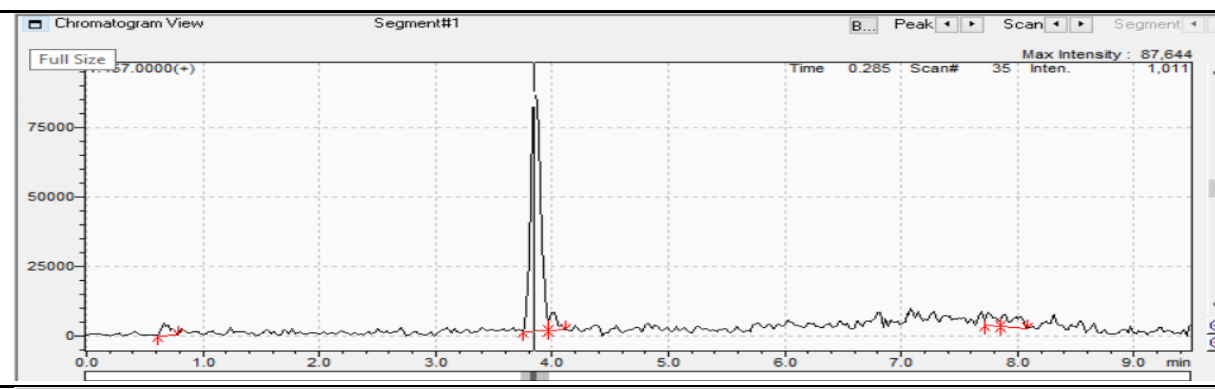	3.854	509127	29.9 %
501	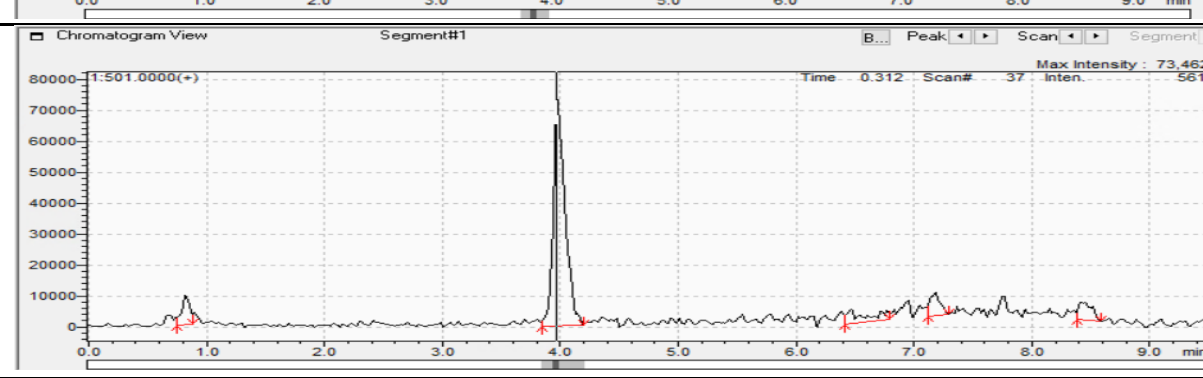	3.976	517735	30.4 %
545	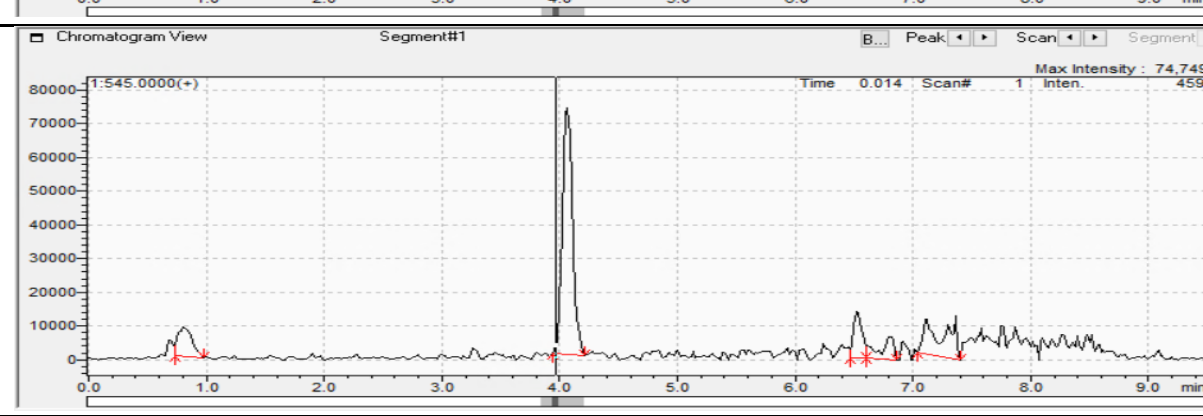	4.062	448192	25.3 %
589	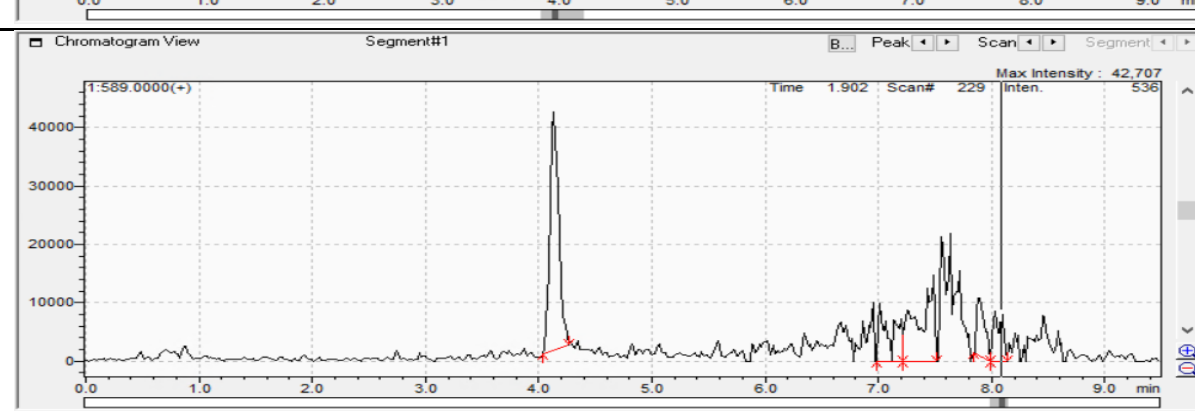	4.132	228668	11.3 %
633	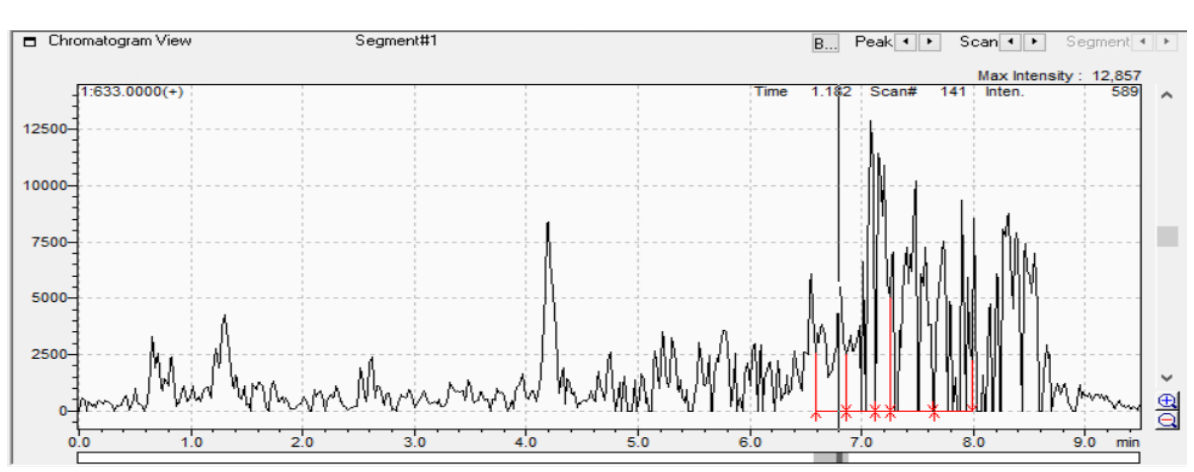	4.201	52901	3.1%
			1703722	

Table S3: Data for pharmacokinetic data (Areas and concentrations)

	Area of Compound:	Area of ETK-III-9	Area of DPI	ETK-II-83/DPI	ETK-III-9/DPI	Conc ETK-II-83	Conc ETK-III-9
0a	26,615,545	33,745	371,120	71.71681666	0.09092746	6765.737	12.124
0b	28,854,832	34,680	355,595	81.14521295	0.09752668	7655.209	13.004
0c	25,550,325	31,327	365,948	69.81955087	0.08560506	6586.750	11.414
0d	24,756,592	29,334	343,329	72.10748874	0.08543991	6802.593	11.392
5a	11,406,574	973,686	341,450	33.40627910	2.85162103	3151.536	380.216
5b	16,758,333	877,475	357,405	46.88891594	2.45512794	4423.483	327.350
5c	11,699,223	1,036,207	364,910	32.06057110	2.83962347	3024.582	378.616
5d	11,231,536	929,851	346,580	32.40676323	2.68293323	3057.242	357.724
10a	6,326,115	1,793,681	328,913	19.23339911	5.45336001	1814.472	727.115
10b	8,176,222	1,457,203	277,120	29.50426530	5.25838265	2783.421	701.118
10c	4,051,556	1,547,281	203,850	19.87518273	7.59029188	1875.017	1012.039
10d	5,323,941	1,527,762	266,082	20.00864771	5.74169617	1887.608	765.559
20a	1,397,892	2,519,727	194,758	7.17758449	12.93773298	677.131	1725.031
20b	1,328,099	2,862,441	244,943	5.42207371	11.68615147	511.516	1558.154
20c	3,576,573	2,803,918	305,254	11.71671133	9.18552419	1105.350	1224.737
20d	1,787,152	3,177,598	363,565	4.91563269	8.74010975	463.739	1165.348
30a	410,976	3,720,547	369,986	1.11078798	10.05591293	104.791	1340.788
30b	1,428,757	3,988,962	354,481	4.03056017	11.25296419	380.242	1500.395
30c	353,818	4,163,756	361,493	0.97876861	11.51822027	92.337	1535.763
30d	495,006	4,178,471	374,789	1.32075915	11.14886243	124.600	1486.515
60a	29,228	6,861,240	349,247	0.08368862	19.64580941	7.895	2619.441
60b	39,052	7,197,149	347,375	0.11242030	20.71867290	10.606	2762.490
60c	28,033	6,862,806	321,668	0.08714886	21.33505975	8.222	2844.675
60d	22,747	7,127,785	333,170	0.06827445	21.39383798	6.441	2852.512
120a	35,763	10,342,214	346,649	0.10316776	29.83483004	9.733	3977.977
120b	36,685	11,886,291	339,295	0.10812125	35.03231996	10.200	4670.976
120c	31,802	10,630,816	343,884	0.09247886	30.91395936	8.724	4121.861
120d	30,947	11,155,739	362,052	0.08547667	30.81253245	8.064	4108.338

Free drug brain binding study

A 48-well rapid equilibrium dialysis (RED) device (Thermo Scientific, USA) was used to determine the free fractions of compounds ETK-II-83 and ETK-III-9 in mouse brain tissue. Mouse brain tissue samples were diluted with four volumes of phosphate-buffered saline (PBS) and homogenized to obtain a uniform brain homogenate. For each RED insert, 495 µL of brain tissue homogenate was added to the sample chamber, followed by 5 µL of 1 mM methanolic working solution of either compound ETK-II-83 or compound ETK-III-9 to achieve the final concentration of 10 µM. A volume of 500 µL PBS was added to the corresponding buffer chamber. The plate was sealed and incubated at 37 °C on an orbital shaker for 4 h to allow equilibration between the chambers. Following incubation, 50 µL was collected from both the buffer and brain homogenate chambers and transferred to microcentrifuge tubes. Matrix matching was performed by adding 50 µL of blank brain homogenate to each buffer sample and 50 µL of blank PBS to each brain homogenate sample to ensure identical matrix composition. Protein precipitation and extraction were carried out by adding 300 µL of chilled methanol containing internal standard (1 µM) to each sample. The mixtures were vortex-mixed, incubated on ice for 30 min, and immediately centrifuged at 14000 ×g for 10 min. The supernatants were transferred to clean tubes and evaporated to dryness under a gentle stream of nitrogen. The dried residues were reconstituted in 150 µL methanol, sonicated, and centrifuged. The samples were subsequently passed through Spin-X filters by centrifugation at 9000 rpm for 2 min, and 100 µL of the filtrate was transferred to autosampler vials for analysis using an LC–MS/MS system (Shimadzu LCMS-8040, Shimadzu, Japan). For detailed analysis description see microsomal stability assay.

The apparent unbound fraction (fu) for each compound was calculated as the ratio of the concentration measured in the buffer chamber to that measured in the corresponding brain

homogenate chamber. The unbound fraction in undiluted brain tissue for each compound was calculated using the appropriate dilution correction equation.[3]

Table S4: Data for Free drug brain binding.

DPI RT=	2.77 min												
ETK-II-83 RT=	4.95 min												
Brain: Day 1 - 02.12.2026													
	Buffer Chamber			Brain Chamber									
Sample	ETK-II-83 Peak Area	DPI Peak Area	ETK-II-83/DPI	ETK-II-83 Area	DPI Peak Area	ETK-II-83/DPI	fu Apparent	unbound % diluted	D	1/D	1/fu Apparent	fu undiluted	unbound % undiluted
1	1,430,480	1,702,610	0.840168917	3,933,299	2,127,735	1.848584998	0.454492987	45.45	6.2	0.16129	2.20025397	0.118461311	11.85
2	1,347,614	1,686,707	0.798961527	3,927,002	1,957,184	2.006455193	0.398195549	39.82	6.2	0.16129	2.51132893	0.09642979	9.64
3	1,523,848	1,651,462	0.922726651	3,224,367	1,787,524	1.803817459	0.511541035	51.15	6.2	0.16129	1.95487738	0.144503664	14.45
A	1,668,551	1,934,163	0.78419117	3,531,281	2,000,608	1.765103908	0.444274791	44.43	6.2	0.16129	2.2508592	0.114216179	11.42
B	1,899,232	1,756,607	0.970390112	3,692,221	1,880,623	1.963296737	0.494265637	49.43	6.2	0.16129	2.02320357	0.136168134	13.62
C	1,753,494	1,720,447	0.980962493	3,399,566	1,824,017	1.863779778	0.526329615	52.63	6.2	0.16129	1.89995009	0.151982831	15.20
													12.70
DPI RT=	2.76 min												2.09
ETK-III-9 RT=	4.37 min												
Brain: Day 1 - 02.12.2026													
	Buffer Chamber			Brain Chamber									
Sample	ETK-III-9 Peak Area	DPI Peak Area	ETK-III-9/DPI	ETK-III-9 Area	DPI Peak Area	ETK-III-9/DPI	fu Apparent	unbound % diluted	D	1/D	1/fu Apparent	fundiluted	% undiluted
1	3,391,520	2,039,810	1.66266466	13,016,115	2,094,741	6.213710907	0.267579983	26.76	6.2	0.16129	3.73720032	0.055646315	5.56
2	3,467,035	1,806,131	1.919592211	11,926,926	1,796,354	6.639518714	0.289116168	28.91	6.2	0.16129	3.45881728	0.061558664	6.16
A	4,338,400	1,810,581	2.396136931	13,084,412	1,887,403	6.932495074	0.345638461	34.56	6.2	0.16129	2.89319654	0.078506378	7.85
B	4,048,061	1,719,465	2.35425612	12,485,893	1,737,093	7.187809173	0.327534589	32.75	6.2	0.16129	3.05311266	0.072836933	7.28
C	3,878,260	1,617,676	2.397426926	14,529,992	1,759,037	8.260196915	0.290238471	29.02	6.2	0.16129	3.44544262	0.06187451	6.19
													6.61
													0.93

DPI RT=	2.78 min								
ETK-II-83 RT=	4.95 min								
Blood: Day 1 - 02.27.2026									
	Buffer Chamber			Brain Chamber					
Sample	ETK-II-83 Peak	DPI Peak Area	ETK-II-83/DPI	ETK-II-83 Area	DPI Peak Area	ETK-II-83/DPI	fu Apparent	unbound %	
1	149,951	1,358,147	0.11040852	3,163,448	1,902,282	1.662975311	0.066392158	6.64	
2	163,254	1,461,305	0.111717951	2,331,778	1,660,065	1.404630542	0.07953547	7.95	
3	160,465	1,422,790	0.112781928	2,892,776	1,656,259	1.746572245	0.064573297	6.46	
A	130,904	1,742,304	0.075132698	2,799,324	1,663,887	1.682400307	0.044658039	4.47	
B	195,714	1,605,541	0.121899098	3,252,879	1,765,140	1.84284476	0.066147242	6.61	
C	160,512	1,537,616	0.104390173	2,834,116	1,741,265	1.627619001	0.064136738	6.41	
								6.42	
DPI RT=	2.77 min							1.12	
ETK-III-9 RT=	4.37 min								
Blood: Day 1 - 02.27.2026									
	Buffer Chamber			Blood Chamber					
Sample	ETK-III-9 Peak Area	DPI Peak Area	ETK-III-9/DPI	ETK-III-9 Area	DPI Peak Area	ETK-III-9/DPI	fu Apparent	% unbound	
1	836,264	1,610,243	0.519340249	10,059,334	1,485,263	6.772762804	0.076680708	7.67	
2	801,318	1,570,880	0.51010771	8,197,569	1,687,925	4.856595524	0.105034012	10.5	
3	1,175,605	1,478,209	0.795290111	10,046,873	1,649,921	6.089305488	0.130604404	13.06	
A	987,069	1,564,734	0.630822236	9,632,781	1,651,060	5.834300995	0.108123019	10.81	
B	771,785	1,510,556	0.510927764	768,404	95,301	8.062916444	0.063367612	6.34	
C	763,141	1,319,632	0.578298344	8,761,446	1,542,748	5.679116745	0.101828923	10.18	
								9.76	
									2.40

References

[1] Fischer, B. D., Schlitt, R. J., Hamade, B. Z., Rehman, S., Ernst, M., Poe, M. M., Li, G., Kodali, R., Arnold, L. A., and Cook, J. M. (2017) Pharmacological and antihyperalgesic properties of the novel alpha2/3 preferring GABA(A) receptor ligand MP-III-024, *Brain Res Bull 131*, 62-69.

[2] Roni, M. S. R., Zahn, N. M., Mikulsky, B. N., Webb, D. A., Mian, M. Y., Knutson, D. E., Guthrie, M. L., Cook, J. M., Stafford, D. C., and Arnold, L. A. (2021) Identification and Quantification of MIDD0301 Metabolites, *Curr Drug Metab 22*, 1114-1123.

[3] Kalvass, J. C., and Maurer, T. S. (2002) Influence of nonspecific brain and plasma binding on CNS exposure: implications for rational drug discovery, *Biopharm Drug Dispos 23*, 327-338.
